# Association between polymorphisms of immune response genes and early childhood caries — systematic review, gene-based, gene cluster, and meta-analysis

**DOI:** 10.1186/s43141-023-00566-x

**Published:** 2023-11-16

**Authors:** P. Aruna, Sneha S. Patil, M. S. Muthu, V. Vettriselvi, Selva Arockiam, R. Kirubakaran, N. Sivakumar

**Affiliations:** 1 Centre for Early Childhood Caries Research, Department of Pediatric and Preventive Dentistry, Sri Ramachandra Institute of Higher Education and Research, Chennai, India; 2 Department of Pediatric and Preventive Dentistry, Indira Gandhi Institute of Dental Sciences, Sri Balaji Vidyapeeth, Pondicherry, India; 3 Department of Environmental Health Engineering, Faculty of Public Health, Sri Ramachandra Institute of Higher Education and Research, Chennai, India; 4 Department of Pediatric and Preventive Dentistry, Dr. D.Y. Patil Dental College and Hospital, Dr. D.Y. Patil Vidyapeeth, Sant-Tukaram Nagar, Pimpri, Pune, India; 5 Centre of Medical and Bio-Allied Health Sciences Research, Ajman University, Ajman, United Arab Emirates; 6 Department of Human Genetics, Sri Ramachandra Institute of Higher Education and Research, Chennai, India; 7 Department of Orthodontics, Meenakshi Ammal Dental College and Hospital, Chennai, India; 8 South Asian Cochrane Network and Centre. Christian Medical College, Vellore, India; 9 Department of Pediatric and Preventive Dentistry, Narayana Dental College and Hospital, Nellore, India

**Keywords:** Genetic variants, Single nucleotide polymorphisms, Immune response genes, Early childhood caries

## Abstract

**Background:**

Early childhood caries is a significant public health concern affecting about 600 million children globally. The etiology of early childhood caries can be explained as an interplay between genetic and environmental factors. Single nucleotide polymorphisms are the most common variations in the human genome. Genetic variations of immune response genes can modify the defense response of the host, and alter the susceptibility to bacterial colonization of the oral cavity and early childhood caries. The aim of this systematic review is to identify genetic variants of immune response genes associated with early childhood caries.

**Results:**

A total of 7124 articles were identified by conducting an elaborate search across various electronic databases and genome-wide association studies databases. Subsequent to exclusion at various stages, fifteen articles qualified to be included into the present review. Risk of bias assessment was done with the Q-genie tool. Quantitative synthesis revealed that the odds ratio for TT and CC genotypes of rs11362 was 1.07 (0.67–1.71) and 1.16 (0.84–1.60), respectively. Gene-based analysis revealed a statistically significant association between variants of tumor necrosis factor-alpha gene and T-cell receptor alpha variable 4 locus with early childhood caries. Gene clustering showed the presence of three functional clusters. To comprehend the protein–protein interaction, the bioinformatic tool of “Search Tools for the Retrieval of Interacting Genes and Proteins” was used. Among the biological processes and the reactome pathways, complement activation through the lectin pathway showed the highest strength of association with early childhood caries. To understand the interaction and functionality of the genes, “gene function prediction using Multiple Association Network Integration Algorithm” was used, which revealed that the genes were linked by physical interaction (39.34%) and through co-expression (34.88%).

**Conclusions:**

Genotype TT of rs7217186 of arachidonate 15-lipoxygenase gene was a risk factor for early childhood caries. Multiple genetic variants of T-cell receptor alpha variable 4 locus and tumor necrosis factor-alpha gene were associated with increased susceptibility to early childhood caries. Polymorphisms of genes regulating the lectin pathway of complement activation can modify the susceptibility to early childhood caries.

**Supplementary Information:**

The online version contains supplementary material available at 10.1186/s43141-023-00566-x.

## Background

Early childhood caries (ECC) is a chronic complex disease affecting children characterized by demineralization of calcified tissues and destruction of organic tissues of teeth. Genetic predisposition to dental caries was suggested by Dr G V Black [1]. Variations observed in the susceptibility to caries, following exposure to the same risk factors, can be explained by the innate genetic factors [2]. Genetic contribution to caries development has been reported to range between 40 and 60% [3–5] with the heritability of caries in primary dentition being greater than the heritability of caries in permanent dentition [5, 6]. Genes regulating amelogenesis, immune response, taste preferences, glucose metabolism, salivary composition, and flow alter the susceptibility to ECC [7]. Saliva plays a crucial role in oral defense mechanisms and certain proteins secreted in saliva contribute to its anti-microbial properties. Single nucleotide polymorphisms (SNPs) of genes encoding these proteins may modify the antimicrobial property of saliva, thus leading to the establishment of cariogenic microflora.

Polymorphisms of genes regulating Immune response can alter the defense response of the host. Lactotransferrin *(LTF*), an iron-binding glycoprotein in mammalian secretions exhibits broad-spectrum antimicrobial activity, participates in inflammation, and regulates the immune response [8]. rs1126478 of the *LTF* gene can influence caries development [8] and genotype AA of rs1126478 displays bioactivities against other acid-producing microbes [9]. Defensin beta 1 (*DEFB1*) gene regulates microbial colonization and polymorphisms in the promoter region of this gene may alter the caries susceptibility [10–12]. Mannose-binding lectin (*MBL2*) plays an important role in innate immunity [13, 14]. Mutations of codon 54 of MBL2 are associated with recurrent infections and autoimmune diseases [15, 16]. Differences in major histocompatibility complex (*MHC*) or human leukocyte antigen (*HLA*) may cause variations in the immune response and influence the susceptibility to ECC [17]. Arachidonate 15-lipoxygenase (*ALOX15*) regulates inflammation and immune response and TT genotype of rs7217186 is a risk factor for ECC [18].

SNPs of immune response genes may alter the immune response, inflammatory reactions, and cytokine production and may modify the susceptibility to ECC. Studies on the association between polymorphisms of genes regulating immune response and dental caries in children have yielded inconsistent results. This systematic review aims to comprehend the association between genetic variants of immune response genes and ECC.

## Methods

### Registration of protocol and reporting guidelines

The systematic review was registered with PROSPERO (International Prospective Register of Systematic Reviews) with protocol number CRD42020179922 and is reported as per the PRISMA (Preferred Reporting Items for Systematic Reviews and Meta-analysis) checklist 2020 [19]. We deviated from the protocol by including only the studies evaluating the polymorphisms of Immune response genes in this review.

### Eligibility criteria

The research question of the present review was to ascertain the polymorphisms of immune response genes associated with ECC. The review followed the PECO framework (1) participants/population: children up to 6 years of age; (2) exposure: SNPs and genetic variants of genes regulating immune response (3) comparison: children without the polymorphisms of immune response genes; (4) outcome being ECC.

Observational studies (cross-sectional, case–control, and cohort design) that assessed the association of SNPs and variations in genes regulating immune response with ECC were included. Studies conducted on animals, case reports, case series, and those not in English were excluded.

### Search strategy

An extensive search was conducted across various electronic databases such as MEDLINE via PubMed, CINAHL via EBSCO, LILACS, Web of Science, SCOPUS, EMBASE, Cochrane Central, Google Scholar, and Opengrey and GWAS (Genome-Wide Association Study) databases from January 2003 (completion of Human Genome Project) till September 2022. The search strategy has been summarized in Supplementary Table 1. The references of the existing reviews were assessed for relevant studies. A hand search of *Journal of Clinical Pediatric Dentistry*, *Journal of Dentistry for Children*, *Pediatric Dentistry*, *International Journal of Paediatric Dentistry*, *European Archives of Paediatric Dentistry*, *Caries Research*, *Pediatric Dental Journal*, *Journal of Indian Society of Pedodontics and Preventive Dentistry*, and *Genetic Epidemiology and American Journal of Epidemiology* were also conducted.

### Selection of studies

The titles and abstracts of the selected studies were screened by two authors (PA and SP) independently and were grouped as included, excluded, and uncertain studies (if the abstract was ambiguous or unavailable). The full texts of the included studies and studies in uncertain categories were evaluated and studies which did not satisfy the eligibility criteria were excluded from the review. Disagreements about the inclusion of the studies were resolved either by consensus or by the third author (MSM). The corresponding author was contacted to elicit any missing, unreported data.

### Data extraction

Two authors (PA and SP) recorded the data independently in a customized data extraction form. Data regarding author’s name, institutional affiliation, journal name, year of publication, study design, ethnicity of participants, chromosome, gene, sample size, SNPs analyzed, genotype and allele frequencies, co-variates evaluated, odds ratio (OR) at 95% confidence intervals (CI) and *p*-value were obtained.

### Assessment of risk of bias

The quality of the included studies was assessed by two authors (PA and SP) using the Q-Genie tool [20]. This tool was designed and validated to evaluate the quality of studies analyzing the genetic association. It is a Likert-type scale, consisting of eleven questions. The maximum score for each question is seven and the minimum score is 1. For studies with a control group, a score < 35 indicates poor quality, in the range of 35–45 indicates moderate quality, and > 45 indicates good quality. Similarly, for studies without control groups, a score < 32 indicates low quality, in the range of 32–40 indicates moderate quality, and greater than 40 indicates good quality. Any difference of opinion was resolved by consensus or by another author (VV).

### Data synthesis

Review Manager statistical software (RevMan 5.4, The Cochrane Collaboration, London, UK) was used to analyze the results of the included studies. The SNPs analyzed across the included studies were scrutinized and their genotype frequencies were collated to generate the forest plots and the pooled OR at 95% CI was calculated to estimate the effect sizes. The inverse-variance method was used to estimate the weight of the study. Heterogeneity was assessed by evaluating the population and study characteristics. The *I*^2^ analysis and chi-square test were conducted to assess heterogeneity between the studies and a random-effects model was used to conduct the meta-analysis.

Plink software and R statistical software were used to perform the gene-based analysis and gene pair-based associations. “BiomaRt” and “BS genome and Homosapiens.UCSC.hg38” packages were used to extract gene coordinates for each corresponding reference SNP cluster ID (RSIDs). The linkage disequilibrium *r*^2^ value was computed using Plink. Fisher’s exact approach, Simes approach, ECS (extended chi-square) approach, GATES (Gene-based Association Test using extended Simes procedure), inverse method, weighted truncated product method (TPM), unweighted truncated product method (TPM), and Adaptive Rank Truncated Product (ARTP) were used to perform gene-based analysis, with an error rate of 0.05. The largest test statistic from all the SNP-based tests in a gene was used as a gene-based test statistic.

Significant association within the immune response gene cluster were determined by gene cluster analysis. “GeneGeneInteR 1.22.0”, “BS genome,” “Biobase,” “Biocgenerics,” “Biocmanager,” and “ARTP2” packages were applied to perform gene-based and gene cluster analysis. Significant gene pairs associated with ECC were determined with LD (Linkage Disequilibrium) attenuating rank sum test. The multiple testing for pathway p values was performed using “Benjamini & Hochberg 1995” with a false detection rate (FDR) threshold set at 0.05 [21].

Enrichment analysis with protein–protein interaction (PPI) network construction was done to evaluate the functional impacts of differentially expressed genes. Search Tools for the Retrieval of Interacting Genes and Proteins (STRING) plot was constructed (https://string-db.org) wherein network nodes represent genes and lines of different colors represent different types of evidence used in predicting associations [22]. Possible gene network association and gene interaction were predicted using geneMANIA (gene function prediction using Multiple Association Network Integration Algorithm). The possible interaction network is predicted using many publicly available datasets on gene–gene and protein–protein interaction networks. GeneMANIA (http://www.genemania.org) is used for prioritizing genes for functional assays. Given a list of query genes, geneMANIA extends the list by including other functionally similar genes from the available genomics and proteomics data [23].

## Results

### Search outcome

**Fig. 1:**
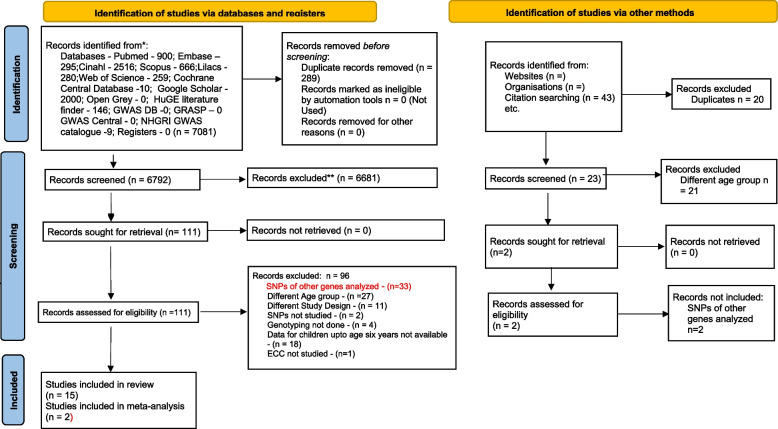
PRISMA flow diagram

A total of 7124 articles were identified after a comprehensive search of databases. Initial screening resulted in the exclusion of 7011 articles, including duplicates. After a full-text screening of the remaining 113 articles, fifteen articles which satisfied the inclusion criteria were included in the present review. The PRISMA flow diagram depicting this is shown in Fig. 1. The included and excluded studies have been tabulated in Supplementary Table 2 and Supplementary Table 3, respectively.

### Description of studies

The studies were published from 2006 to 2022. The number of participants varied from 37 [24] to 1005 [25]. The studies were performed on diverse population groups such as Chinese, Turks, Norwegians, Polish, Caucasians, Hispanic Whites and Blacks, Non-Hispanic Whites and Blacks, Brazilians, Iranians, and Saudi Children. Eight studies were designed as case–control studies [25–32], two were cross-sectional [17, 18] and five were cohort studies [24, 33–36].

Thirteen studies assessed the association between 23 SNPs of seven genes regulating immune response with ECC in 4240 children; one study evaluated the relationship between four SNPs of T-cell receptor alpha variable 4 (*TRAV4*) locus and ECC in 176 children and one study analyzed the association between various alleles of Human Leucocyte Antigen (*HLA*) and ECC in seventy-nine participants. Six polymorphisms of the *LTF* gene were analyzed of which two were coding sequence variants and four were intronic variants. Eight polymorphisms of Lactoperoxidase (*LPO*) gene were evaluated of which six polymorphisms were intronic and two were Upstream Transcript Variants. Three Upstream transcript variants of *DEFB1* and two intronic polymorphisms of *ALOX15* were assessed. One coding sequence variant and one Upstream transcript variant of the *MBL2* gene, one coding sequence polymorphism of mannose-binding lectin-associated serine protease 2 (*MASP2*) and a single upstream transcript variant of tumor necrosis factor-alpha (*TNF-α*) were evaluated for the relationship to ECC.

### Quality assessment

The fifteen included studies scored in the range of 36 to 50 with the Q-Genie tool. Eleven studies were of good quality and four studies were of moderate quality [17, 24, 27, 28]. The quality assessment has been tabulated in Table 1.

**Table 1:** Risk of bias assessment of included studies

S. No	Author and Year	Ratio nale for study	Selection and definition of outcome of interest	Selection and comparability of comparison groups	Technical classification of the exposure	Non-technical classification of the exposure	Other sources of bias	Sample size and power	A priori planning of analyses	Statistical methods and control for confounding	Testing of assumptions and inferences for genetic analyses	Appropriateness of inferences drawn from results	Final Score	Qualit y of the study
1	Bagherian et al., 2008 [17]	6	5	5	3	1	1	1	5	5	3	6	41	Moder ate
2	Olszowski et al., 2012 [26]	6	5	5	5	1	1	5	5	5	5	6	49	Good
3	Briseño-Ruiz et al., 2013 [33]	6	5	5	4	1	1	3	6	6	4	6	47	Good
4	Krasone et al., 2013 [24]	6	5	5	1	1	1	1	6	1	3	6	36	Moderate
5	Yang et al., 2013 [27]	6	5	5	5	1	1	1	6	2	3	6	41	Moder ate
6	Mubayrik et al., 2014 [28]	6	6	5	4	1	1	1	5	4	5	6	44	Moder ate
7	Stanley et al., 2014 [34]	6	5	6	5	1	1	3	5	6	5	6	49	Good
8	Abbasoğlu et al., 2015 [18]	6	6	6	1	1	4	4	5	5	4	6	48	Good
9	Lips et al., 2017 [35]	6	6	5	5	1	1	1	6	6	5	6	48	Good
10	Wang et al., 2017 [25]	6	5	5	6	1	1	4	6	6	2	6	48	Good
11	Wang et al., 2018 [29]	6	5	5	5	1	1	6	6	6	3	6	50	Good
12	Weber et al., 2018 [36]	6	5	6	5	1	1	1	5	6	6	6	48	Good
13	Al-Marshad et al., 2021 [30]	6	6	5	3	1	1	5	5	5	6	6	49	Good
14	Zaorska et al., 2021 [31]	6	6	6	4	1	1	1	6	6	6	6	49	Good
15	Wu et al., 2022 [32]	5	6	6	4	1	1	5	5	5	5	6	49	Good

### Quantitative synthesis

**Fig. 2:**
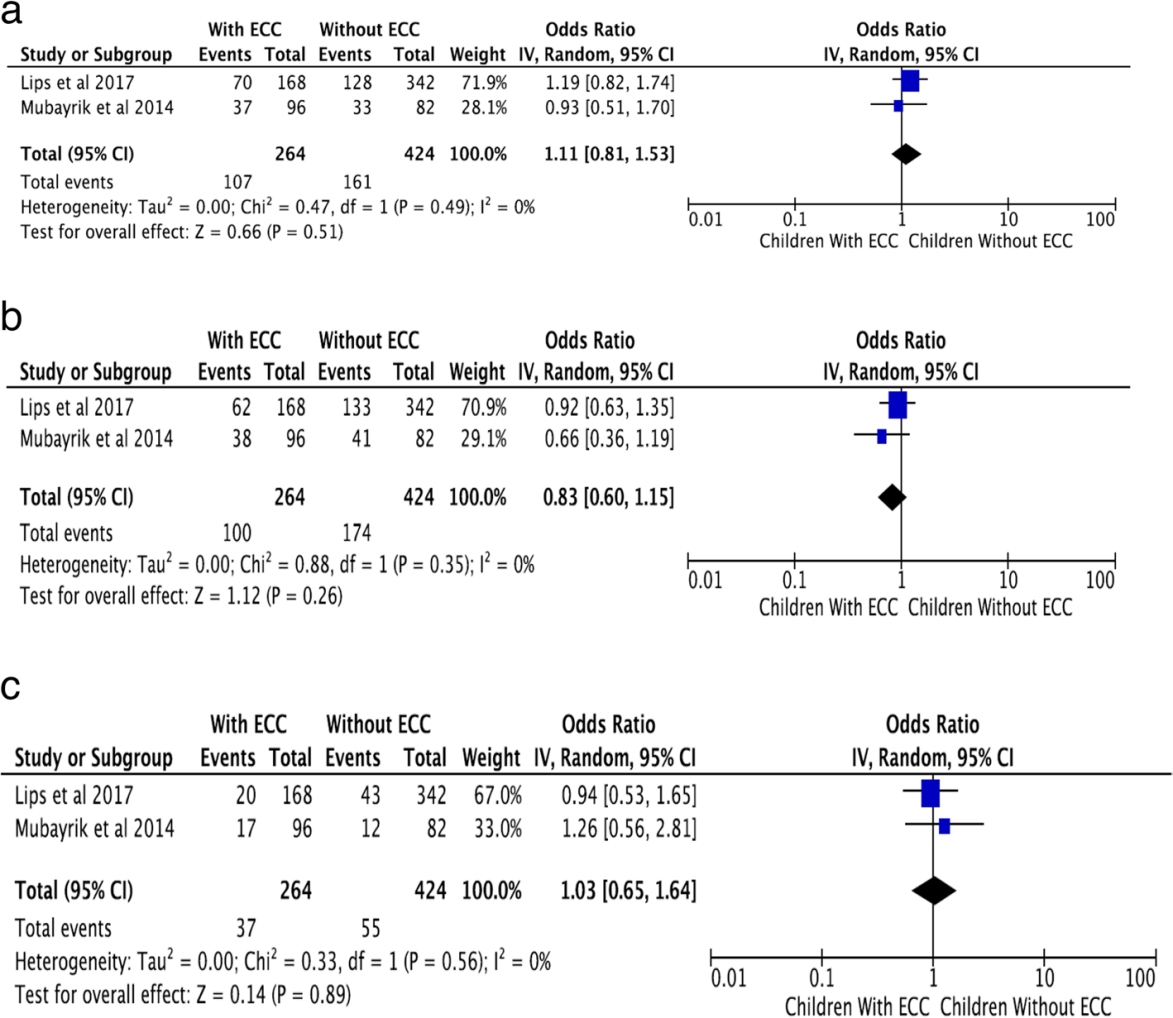
**a**–**c** Forest plots of CC, CT, and TT genotypes of rs11362

Among the fifteen included studies, two studies that analyzed one SNP have been included in the meta-analysis. Studies with various designs were pooled together as the genetic association was analyzed using the inverse variance method with random-effect models. The TT genotype of the polymorphism rs11362 of *DEFB1* displayed an OR of 1.03; 95%CI ranging from 0.65 to 1.64 and an insignificant *P* value of 0.89. The CC genotype revealed an OR of 1.11 and 95% CI ranging from 0.81 to 1.53 and a *P* value of 0.51 which was not statistically significant. The heterozygous genotype CT returned an OR of 0.83; 95% CI (0.60–1.15) and a *P* value of 0.26, which lacked statistical significance. The forest plots generated are shown in Fig. 2. Meta-analysis was not performed for rs1126478 as the same data was reported in two studies with a difference in the phenotype [24, 28]. Meta-analysis could not be performed for the other variants as the genotype frequencies were not reported.

Six polymorphisms of *LTF* (rs1126478, rs1126477, rs2269436, rs743658, rs4547741, rs17078878) eight polymorphisms of *LPO* (rs8178350, rs7209537, rs17762644, rs8178281, rs8178290, rs8178307, rs8178329, rs3744093) two variants of *DEFB1* (rs1799946, rs1800972) and *ALOX15* (rs2619112, rs7217186), three SNPs of *MBL2* (rs1800450, rs7096206, rs11003125), four variants of *TRAV4* locus (rs1997532, rs8011979, rs7150049, rs1997533), single SNP of *MASP2* (rs72550870), and *TNF-α* (rs1800629) were evaluated in various studies. However, a meta-analysis could not be performed as the genotype frequencies were not reported.

### Gene-based and gene cluster analysis

Gene-based analysis of the four variants in the *TRAV4* locus revealed a significant association with ECC as reflected by the *P* values of the various statistical approaches — Fischer’s exact approach (*P* = 0.0026688), Simes approach (*P* = 0.002), GATES (*P* = 0.042), inverse method (*P* = 0.007375), ECS (*P* = 0.042), weighted TPM (*P* = 0.00218), unweighted TPM (*P* = 0.001962), ARTP (*P* = 0.001744). The polymorphism rs1800629 of *TNF-α* also revealed a statistically significant association with ECC under all the above-mentioned statistical approaches except GATES (*P* = 0.0805) and ECS (*P* = 0.0805). The results of the gene-based analysis are depicted in Table 2. Gene cluster analysis did not reveal a statistically significant association between polymorphisms of immune response gene cluster and incidence of ECC (Table 3).

**Table 2:** Gene-based analysis of immune response genes

Symbol	Chromosome	Start position	Group	SNP	Position	Gene Feature	Meta-P	Fischers	Simes	GATES^a^	ECS^b^	Inverse	Weighted TPM^c^	Unweighted TPM	ARTP^d^
TRAV4	14	21677306	Protein-coding gene	rs1997533	21729284	Intronic	0.0005	0.0026688	0.002	0.042	0.042	0.007375	0.00218	0.001962	0.001744
				rs8011979	21733619	Intronic	0.009								
				rs7150049	21733607	Intronic	0.01								
				rs1997532	21729203	Intronic	0.01								
TNF-*α*	6	31554976	Protein-coding gene	rs1800629	31557394	Exonic	0.023	0.026688	0.023	0.0805	0.0805	0.023	0.025185	0.0226665	0.015111
		31654731		rs1800629	31651559	Upstream	0.023								
		31644460		rs1800629	31651559	Upstream	0.023								
		31606804		rs1800629	31614702	Intronic	0.023								
		31557048		rs1800629	31562660	Upstream	0.023								
				rs1800629	31565480	Upstream	0.023								
				rs1800629	31557394	Exonic	0.023								
ALOX15	17	4637803	Protein-coding gene	rs2619112	4632090	Upstream	0.04	0.06672	0.05	0.116666667	0.116666667	0.045	0.05475	0.049275	0.0438
				rs7217186	4636097	Intronic	0.05								
MBL2 -	10 -	52750647 -	Protein-coding gene	rs7096206	52771925	Intronic	0.023	0.092046	0.069	0.1449	0.1449	0.1365	0.075831	0.0682479	0.0606648
				rs1800450	52771475	Intronic	0.25								
DEFB1	8	6873390	Protein-coding gene	rs1800972	6877901	Upstream	0.02	0.160128	0.12	0.200307692	0.200307692	0.2693333	0.13164	0.118476	0.105312
				rs1799946	6877871	Upstream	0.074								
				rs1799946	6877909	Upstream	0.314								
				rs11362	6877839	Upstream	0.314								
				rs1800972	6877863	Upstream	0.432								
				rs11362	6877877	Upstream	0.462								
LTF	3	46458006	Noncoding RNA	rs4547741	46458968	ncRNA	0.036	0.1894848	0.142	0.208631956	0.208631956	0.6392	0.155774	0.1401966	0.1090418
				rs1126478	46459723	ncRNA	0.062								
				rs17078878	46459610	ncRNA	0.382								
		46449105	Protein-coding gene	rs743658	46446997	Upstream	0.08								
				rs2269436	46445762	Upstream	0.396								
LPO	17	58254688	Protein-coding gene	rs7209537	58250853	Downstream	0.093	0.38654232	0.289675	0.337295231	0.337295231	0.09974	0.317773475	0.285996128	0.25421878
				rs3744093	58415439	Intronic	0.1102								
		58227333	Protein-coding gene	rs7209537	58250853	Downstream	0.093								
				rs8178329	58249160	ncRNA	0.1183								
				rs8178281	58239832	Downstream	0.2874								
HLA DRB1	6 6	32358286	Noncoding RNA	rs3763305	32359153	Downstream	0.199	0.3796368	0.2845	0.338745098	0.338745098	0.256	0.3120965	0.28088685	0.21846755
		32361115	Protein-coding gene	rs3763305	32359153	Downstream	0.199								
				rs3763305	32377195	Upstream	0.37							0.001962	0.001744

^a^Gene-based Association Test using extended Simes procedure

^b^Extended chi-square

^c^Truncated product method

^d^Adaptive Rank truncated Product

**Table 3:** Gene cluster analysis of immune response genes

Gene cluster	Fischers	Simes	GATES^a^	ECS^b^	Inverse	Weighted TPM^c^	Unweighted TPM	ARTP^d^
Immune response gene cluster	0.15027	0.112952	0.17536	0.175362	0.16732	0.12389	0.11151	0.09289

^a^Gene-based Association Test using extended Simes procedure

^b^Extended chi-square

^c^Truncated product method

^d^Adaptive Rank truncated Product

### Enrichment analysis

**Fig. 3:**
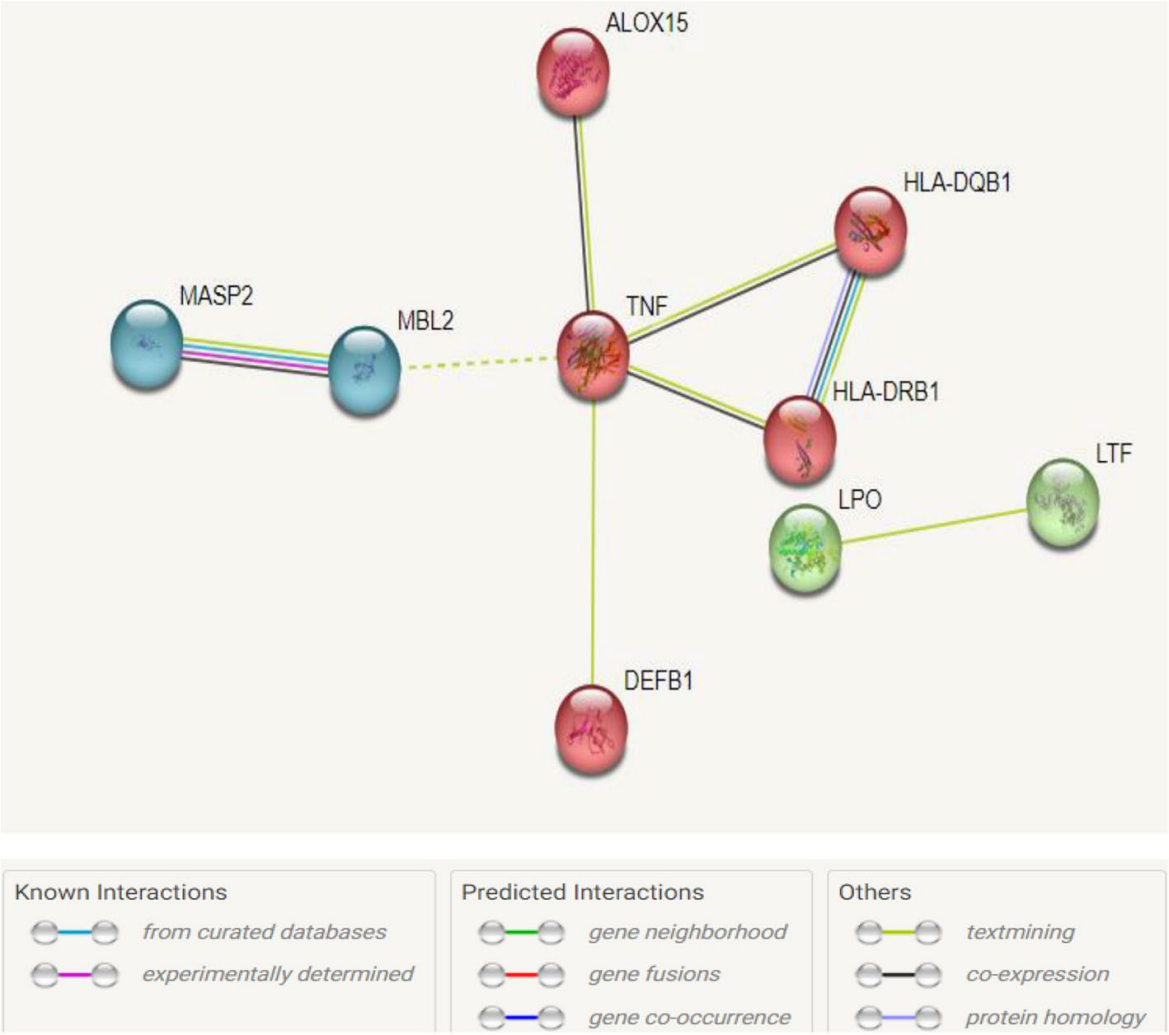
STRING plot showing clustering of genes into 3 clusters and their interaction

The constructed enrichment network consisted of nine nodes (differentially expressed genes) and eight edges with a strength of interaction score set at > 0.8. The PPI enrichment coefficient, average node degree, and average local clustering coefficient were < 2.35e − 06, 1.78, and 0.789, respectively. With the number of pre-defined clusters being three, the network was constructed with the Kmeans hierarchical clustering algorithm. The generated network revealed that *ALOX15*, *DEFB1*, *HLA-DQB1*, *HLA-DRB1*, and *TNF-α* constituted one cluster, *LTF* and *LPO* defined the second cluster, and *MBL2* and *MASP2* were in the third cluster. The enrichment analysis with the three clusters is depicted in Fig. 3.

**Fig. 4:**
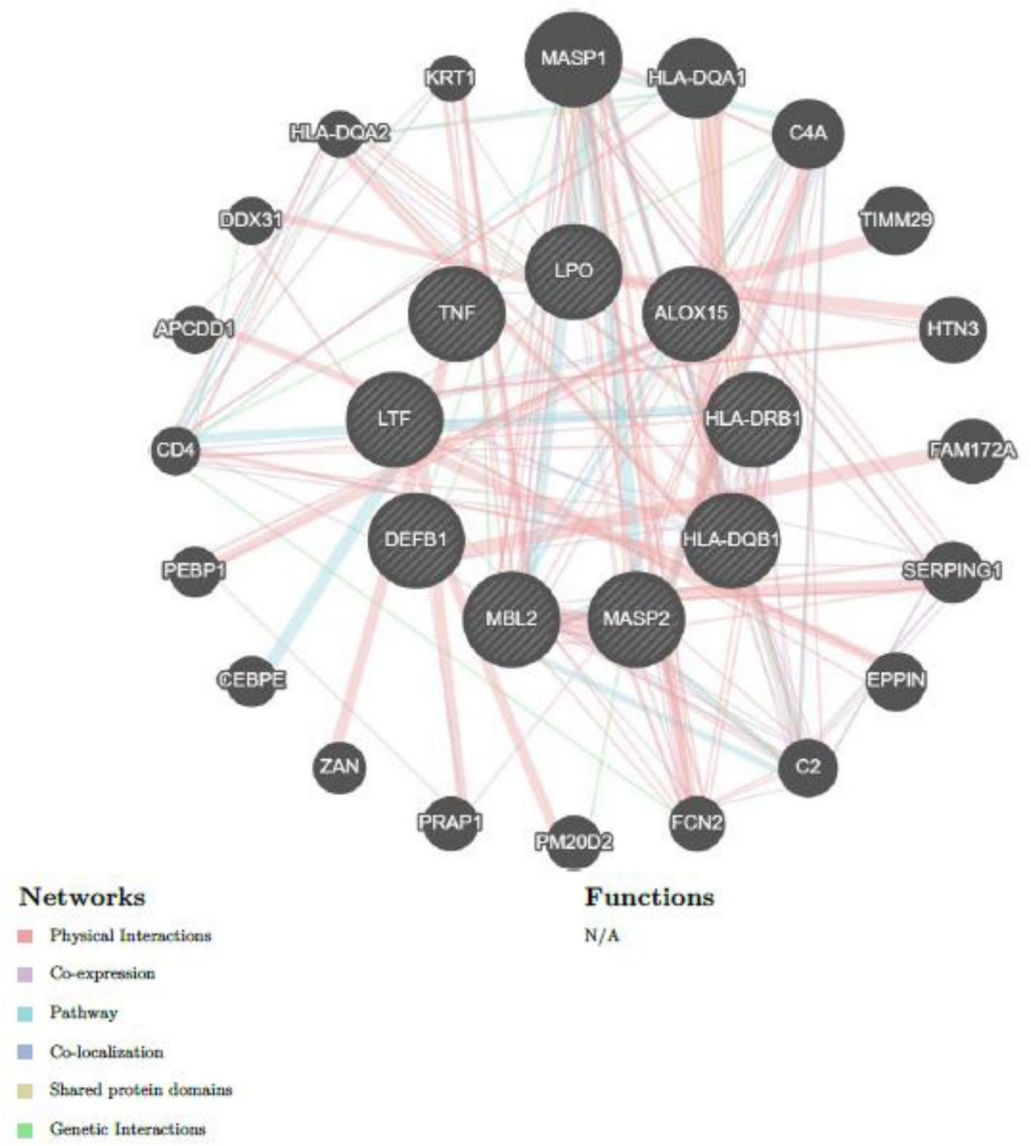
geneMANIA plot showing the interaction between various genes

Biological pathways under the enrichment analysis revealed that seven genes (*LTF*, *DEFB1*, *HLA-DRB1*, *MBL2*, *HLA-DQB1*, *MASP2*, *TNF-α*) were associated with humoral immune response. Four genes *HLA-DRB1, MBL2*, *HLA-DQB1*, and *MASP2* were related to humoral immune response mediated by circulating immunoglobulin. Genes *LTF*, *DEFB1*, *HLA-DRB1*, *MBL2*, *HLA-DQB1*, *MASP2* were linked to Innate Immune response. The lectin pathway of complement activation was mediated by *MBL2* and *MASP2.* Three genes *MBL2*, *TNF-α*, and *ALOX15* played a role in the regulation of phagocytosis whereas *LTF* and *DEFB1* were associated with the innate immune response of the mucosa. Two genes *TNF- α*, *ALOX15* were associated with positive regulation of heterotypic cell–cell adhesion. *HLA-DRB1* and *TNF- α* were linked to the regulation of inflammatory response to antigenic stimulus. *HLA-DRB1* and *HLA-DQB1* were associated with *MHC* class II receptor activity under the molecular pathways. With respect to the cellular component, two genes *HLA-DRB1* and *HLA-DQB1* were associated with the *MHC* class II protein complex, integral component of the lumenal side of endoplasmic reticulum membrane, clathrin-coated endocytic vesicle membrane; *HLA-DRB1*, *HLA-DQB1*, *TNF-α*, and *ALOX15* were associated with the side of the membrane and seven genes *LTF*, *LPO*, *DEFB1*, *HLA-DRB1*, *MBL2*, *MASP2*, and *TNF-α* were related to extracellular space. Reactome pathways revealed that *MBL2* and *MASP2* were related to the lectin pathway of complement activation; *HLA-DRB1* and *HLA-DQB1* were associated with translocation of *ZAP-70* to immunological synapse, phosphorylation of *CD3* and *TCR* zeta chains, and generation of second messenger molecules and with *PD-1* signaling. Wiki pathways showed that *HLA-DRB1* and *TNF-α* were related to Cytokines and inflammatory response and *LTF* and *TNF-α* genes were associated with *LTF* danger signal response pathway. Protein Domains Pfam and SMART (Simple Modular Architecture Research Tools) displayed *HLA-DQB1* and *HLA-DRB1* were associated with Class II histocompatibility antigen, beta domain. Among the biological processes associated with ECC, the lectin pathway of complement activation had the highest strength of association of 2.69 (false discovery rate of 0.0055), and regulation of inflammatory response to antigenic stimulus had a strength of association of 2.21(false discovery rate of 0.0227). Among the reactome pathways, the lectin pathway of complement activation had a strength of association of 2.79 (false discovery rate of 0.0073). Among the protein domains, class II histocompatibility antigen, beta domain had a strength of association of 2.68 with a false discovery rate of 0.0075. This is depicted in Table 4. GeneMANIA plot revealed that the genes which were prioritized are Mannan binding lectin serine peptidase 1 (*MASP1*), *HLADQA1*, *HLADQA2*, translocase of inner mitochondrial membrane 29 (*TIMM29*), family with sequence similarity 172 member A (*FAM172A*), histatin 3 (*HTN3*), complement (C4A), serpin family G member 1 (*SERPING1*), complement C2, epididymal peptidase inhibitor *(EPPIN*), peptidase M20 domain containing 2 (*PM20D2*), proline-rich acidic protein 1 (*PRAP1*), ficolin 2 (*FCN2*), zonadhesin (*ZAN*), DEAD-box helicase 31 (*DDX31*), APC down-regulated 1 (*APCDD1*), phosphatidylethanolamine binding protein 1 (*PEBP1*), CCAAT enhancer binding protein epsilon (*CEBPE*), keratin 1 (*KRT1*), trafficking protein particle complex 2 (*TRAPPC2*), N-myristoyltransferase 2 (*NMT2*), and carboxyl ester lipase (*CEL*). GeneMANIA plot revealed that these genes were linked by physical interaction at 39.34%, co-expression at 34.88%, pathways at 20.08%, co-localization at 2.94%, and shared protein at 2.18%. This is depicted in Fig. 4*.*

**Table 4:** Interactions of commonly mutated immune response genes Indicating biological process and biological pathways

**Biological process**	**GO term**	**Strength**	**False discovery rate**
Complement activation, lectin pathway	GO:0001867	2.64	0.0055
Positive regulation of heterotypic cell–cell adhesion	GO:0034116	2.43	0.0108
Humoral immune response	GO:0002445	2.3	8.07e-06
Innate immune response in mucosa	GO:0002227	2.22	0.0217
Regulation of inflammatory response to antigenic stimulus	GO:0002861	2.21	0.0227
**Molecular pathways (Gene Ontology)**			
MHC class II receptor activity	GO:0032395	2.68	0.0334
**Cellular component Gene Ontology**			
MHC class II protein complex	GO:0042613	2.49	0.0382
Integral component of lumenal side of endoplasmic reticulum	GO:0071556	2.22	0.0438
Clathrin-coated endocytic vesicle membrane	GO:0030669	2.09	0.0438
Side of membrane	GO:0098552	1.21	0.0438
Extracellular space	GO:0005615	0.68	0.0438
**Reactome pathway**s			
*Description*	*Pathway*	*Strength*	*False discovery rate*
Lectin pathway of complement activation	HSA-166662	2.79	0.0073
Translocation of ZAP-70 to Immunological synapse	HSA-202430	2.46	0.0163
Phosphorylation of CD3 and TCR zeta chains	HSA-202427	2.38	0.0163
PD-1 signaling	HSA-389948	2.36	0.0163
Generation of second messenger molecules	HSA-202433	2.18	0.0209
**Wiki pathways**			
LTF danger signal response pathway	WP4478	2.36	0.0089
Cytokines and inflammatory response	WP530	2.22	0.0120
**Protein domains (Pfam)**			
Class II histocompatibility antigen, beta domain	Domain PF00969	2.68	0.0355
**Protein domains (SMART)**			
Class II histocompatibility antigen, beta domain	Domain SM00921	2.68	0.0075

## Discussion

The etiology of ECC can be explained as an interplay of environmental and genetic factors [37]. This systematic review assessed the association between polymorphisms of immune response genes and ECC. Polymorphisms in the coding region as well as in the non-coding regions of the genes regulating the immune response were analyzed to understand their effect on susceptibility to ECC. SNPs in the coding region can change the encoded protein and those in the non-coding region can alter the transcription site and gene expression, thereby altering the predisposition to ECC [38]. Alternate transcription may also result in the synthesis of isoforms of various proteins, thus modifying the susceptibility.

SNPs are the most common variations in the human genome and when analyzing genetic association in complex diseases, a change of a single nucleotide may not always yield results of observable significance. But polymorphisms across the gene can modify the susceptibility significantly. Hence a gene-based analysis identifying the genetic variation across the gene can aid in understanding the alteration in susceptibility as the structure, function, and position of the gene are highly consistent across the individuals with or without the observed phenotype. Differences/inconsistencies arising due to changes in the population sub-structure can also be addressed with this gene-based analysis rather than allele-based association. Clustering helps in grouping of the genes based on similar patterns of gene expression and function. This helps in identifying genes associated with particular biological pathways and evaluation of variation across all such genes can significantly contribute to understanding the genetic underpinnings of the disease.

We deviated from the protocol by including databases Web of Science, SCOPUS, EMBASE, and Google Scholar to increase the specificity of search. Due to the voluminous nature of the included studies, only those studies evaluating the relationship between genetic variants of Immune response genes and ECC were included into the present review. To the best of our knowledge, this is the first systematic review to identify the polymorphisms of immune response genes associated with ECC and to conduct a gene-based and gene cluster analysis to comprehend their effect on ECC.

In this present review, the levels of beta-defensin 1 in the saliva were higher in children without caries [35] and quantitative synthesis revealed that genotype CC of rs11362 could be more in affected children. Genotype TT of rs7217186 of *ALOX15* was a risk factor for ECC [32]*.* The frequency of allele DRB1*04 of HLA was increased in patients with active carious lesions [17]. G variant of rs7096206 of the *MBL2* gene is associated with reduced protein levels in the serum, thus increasing the susceptibility to infections [39, 40], and was a risk factor for ECC in Polish children [25]. Mutant genotype GAC of codon 54 was higher in patients with ECC [26]*.* However, gene-based analysis of the variants of *DEFB1* and *MBL2* did not reveal statistical significance in contrast to findings of meta-analysis by Chistni et al. where *MBL2* was reported to be associated with increased caries experience [41]. MBL-associated serine Protease 2 *(MASP2)* cleaves C2 and C4 to generate C3 convertase in the lectin pathway of the complement system (https://www.ncbi.nlm.nih.gov/gene/10747) [42]. Enrichment analysis revealed that the biological process of the lectin pathway of complement activation had the highest strength of association with ECC followed by humoral immune response and innate immune response of the mucosa, thus indicating that genes involved in these biological processes may affect ECC susceptibility.

The genotype CT of rs4547741 of *LTF* was found to be protective against ECC in Turkish children [32]. However, Al-Marshad et al., Wu et al., and Zaorska et al., did not find any association with ECC [29–31]. Other polymorphisms of *LTF* (rs1126477, rs1126478, rs2269436, rs743658, rs17078878) were not reported to be associated with altered susceptibility to caries [24, 28, 30, 31, 43]. However, gene-based analysis of the *LTF* gene revealed that multiple polymorphisms of the *LTF* gene were significantly associated with caries [44]. This is in contrast to the findings of the present review and could be accounted by differences in the study populations of the included primary studies. Elevated levels of *TNF-α* had been detected in saliva samples of patients with caries. rs1800629 of *TNF-α* is associated with systemic inflammation, and auto-immune diseases and elevated levels were observed in children with caries as a response of the host to pathological stimulus [45]. AG genotype of SNP rs1800629 of *TNF-α* was protective against ECC [28]. G alleles of rs1997533, rs7150049, and T Alleles of rs8011979 and rs1997532 in *TRAV4* locus were associated with low caries experience in Turkish children and mRNA of *TRAV4* is expressed to a greater extent in children with lesser caries experience [33]. Gene-based analysis revealed a statistically significant association between variants of *TRAV4* locus and *TNF-α* with ECC.

The main limitations of this review are that of missing data due to which only two studies were included in the meta-analysis. Despite the heterogeneity being minimal (*I*^2^ = 0), the authors preferred to use the random-effects model, as it accounts for both within-study and between-study variance and is more conservative as it yields a wider confidence interval. Meta-analysis could not be performed for rs1126478 as the same data set was reported in two different studies with a different criterion for the observed phenotype. Certain studies did not have a control group and divided the study subjects into children with low and moderate caries and those with high caries [25]. If the observed phenotype was evaluated uniformly, the effect of the polymorphisms assessed on the carious phenotype may have been different. Most of the studies analyzed the phenotype using the DMFT/deft index. The evaluation of white spot lesions also is to be considered as they are the initial signs of demineralization and disease.

The relationship between the immune response of a host to an antigen is dynamic and can change as per the age of the patients and the dentition. Genes affecting susceptibility to caries differ between primary and permanent dentitions and the direction of association can also change between primary and permanent dentitions. Hence, longitudinal studies can result in more precise phenotypic characterization by assessing the gene-time interaction and aid in understanding the genetic underpinnings of the observed phenotype.

## Conclusions

This review revealed that polymorphisms of *TNF-α*, *ALOX15*, *TRAV4* locus, and alleles of *HLA-DRB1* can modify susceptibility to ECC. Genotype TT of polymorphism rs7217186 of *ALOX15* increased the susceptibility to ECC. Polymorphisms of genes regulating the lectin pathway of complement activation can alter the susceptibility to ECC. Quantitative Synthesis of TT and CC genotypes of rs11362 yielded OR greater than one. However, this has to be interpreted with caution as this evidence is not sufficient to state that rs11362 is a risk factor for ECC. The marginally higher OR suggests that the likelihood of these variants being associated with ECC may be higher which can be corroborated with studies being conducted on more number of individuals. Hence, studies with larger sample size, evaluation of the epigenetic mechanisms, transcriptomics, metabolomics, gene–gene interactions, and protein–protein interaction may aid in understanding the effect of genetic variants of immune response genes and ECC. Application of various BioInformatics tools contributes to understanding the genetic interaction and association. More studies evaluating the polymorphisms of functional significance in these immune response genes can aid in understanding their effect on ECC susceptibility and contribute towards “Personalized and Precision Dentistry.”

### Supplementary Information

**Additional file 1: Supplementary Table 1.** Search Strategy. **Supplementary Table 2.** Table of characteristics of Included studies. **Supplementary Table 3.** Table of Characteristics of Excluded Studies.

## Data Availability

Supplementary file is available.

## Abbreviations

ALOX15: Arachidonate 15-lipoxygenase
ARTP: Adaptive Rank Truncated Product
DEFB1: Defensin beta 1
DMFT/deft: Decayed missing filled teeth/decayed extracted filled teeth
ECC: Early childhood caries
FDR: False detection rate
GATES: Gene-based Association Test using Extended Simes Procedure
geneMANIA: Gene function prediction using Multiple Association Network Integration Algorithm
GWAS: Genome-Wide Association Study
HLA: Human leukocyte antigen
HLA-DQB1: Human leukocyte antigen major histocompatibility complex, class II, DQ beta 1
HLA-DRB1: Human leukocyte antigen major histocompatibility complex, class II, DR beta 1
LD: Linkage disequilibrium
LPO: Lactoperoxidase
LTF: Lactotransferrin
MASP2: Mannose-binding lectin-associated serine protease 2
MBL2: Mannose-binding lectin 2
MHC: Major histocompatibility complex
PECO: Participants, Exposure, Comparison, Outcome
PPI: Protein-protein interaction
PRISMA: Preferred Reporting Items for Systematic Reviews and Meta-Analysis
RSIDs: Reference SNP cluster ID
SNPs: Single nucleotide polymorphisms
STRING: Search Tools for Retrieval of Interacting Genes and Proteins
TPM: Truncated product method
C2: Complement 2
C3: Complement 3
C4: Complement 4
TNF-α: Tumor necrosis factor-alpha
TRAV4: T-cell receptor alpha variable 4
ZAP-70: Zeta chain of T cell receptor-associated protein kinase 70
CD3: Cluster of differentiation 3
TCR: T cell receptor
PD-1: Programmed death 1
SMART: Simple Modular Architecture Research Tool
MASP1: Mannan-binding lectin serine peptidase 1
TIMM29: Translocase of inner mitochondrial membrane 29
FAM172A: Family with sequence similarity 172 member A
HTN3: Histatin 3
C4A: Complement 4A
SERPING1: Serpin family G member 1
EPPIN: Epididymal peptidase inhibitor
PM20D2: Peptidase M20 domain containing 2
PRAP1: Proline-rich acidic protein 1
FCN2: Ficolin 2
ZAN: Zonadhesin
DDX31: DEAD-box helicase 31
APCDD1: APC down-regulated 1
PEBP1: Phosphatidylethanolamine binding protein 1
CEBPE: CCAAT enhancer binding protein epsilon
KRT1: Keratin1
TRAPPC2: Trafficking protein particle complex 2
NMT2: N-myristoyltransferase 2
CEL: Carboxyl ester lipase
