# Supplementary material for: Association between polymorphisms of immune response genes and early childhood caries — systematic review, gene-based, gene cluster, and meta-analysis
Source: J Genet Eng Biotechnol. 2023 Nov 16;21:124. doi: 10.1186/s43141-023-00566-x (PMC10654314; doi:10.1186/s43141-023-00566-x)
Supplement: Supplementary file 1 — Additional file 1: Supplementary Table 1. Search Strategy. Supplementary Table 2. Table of characteristics of Included studies. Supplementary Table 3. Table of Characteristics of Excluded Studies. [file 43141_2023_566_MOESM1_ESM.docx]

*Association between Polymorphisms of immune response genes and Early childhood Caries – A Systematic Review, Gene-based, Gene cluster and Meta-analysis*

Supplementary Material

Supplementary Table 1 – Search Strategy

| PUBMED | (("dental caries"[MeSH Terms] OR ("dental"[All Fields] AND "caries"[All Fields]) OR "dental caries"[All Fields]) OR ("child-hood"[All Fields] AND ("dental caries"[MeSH Terms] OR ("dental"[All Fields] AND "caries"[All Fields]) OR "dental caries"[All Fields] OR "caries"[All Fields]))) OR (("infant"[MeSH Terms] OR "infant"[All Fields]) AND ("dental caries"[MeSH Terms] OR ("dental"[All Fields] AND "caries"[All Fields]) OR "dental caries"[All Fields] OR "caries"[All Fields])) AND (("polymorphism, single nucleotide"[MeSH Terms] OR ("polymorphism"[All Fields] AND "single"[All Fields] AND "nucleotide"[All Fields]) OR "single nucleotide polymorphism"[All Fields] OR ("single"[All Fields] AND "nucleotide"[All Fields] AND "polymorphisms"[All Fields]) OR "single nucleotide polymorphisms"[All Fields]) OR ("polymorphism, genetic"[MeSH Terms] OR ("polymorphism"[All Fields] AND "genetic"[All Fields]) OR "genetic polymorphism"[All Fields] OR "polymorphisms"[All Fields])) OR "genetic"[All Fields]) AND variants[All Fields]) AND (((("genes"[MeSH Terms] OR "genes"[All Fields])))) |
| --- | --- |
| CINAHL | Childhood Caries OR Infant Caries OR Early Childhood Caries AND Single Nucleotide Polymorphisms OR polymorphisms OR genetic variants AND enamel formation genes OR Enamel genes OR Amelogenesis genes |
| LILACS | Caries AND dental OR Childhood Caries AND Single Nucleotide polymorphisms OR Genetic variants AND Enamel genes OR Enamel formation genes OR Amelogenesis Genes |
| EMBASE | (Dental Caries) AND (Single Nucleotide Polymorphisms OR polymorphisms OR genetic variants) AND (Enamel genes OR Enamel formation genes OR Amelogenesis genes)) |
| Web of Science | All Fields (Dental Caries OR Caries OR Infant Caries OR Childhood Caries) AND all fields (genetic variants OR genetic Polymorphisms OR Single Nucleotide Polymorphisms) AND All fields (Enamel formation Genes OR Enamel genes OR Amelogenesis genes) |
| SCOPUS | (ALL (dental AND caries) AND TITLE-ABS-KEY (single AND nucleotide AND polymorphisms) OR TITLE-ABS-KEY (enamel formation genes OR Amelogenesis genes)) |
| Cochrane Central | "dental caries" in Title Abstract Keyword AND “enamel formation genes” in Title Abstract Keyword AND “polymorphisms” in Title Abstract Keyword OR “genetic variants” in Title Abstract Keyword |
| GWAS databases | Dental caries AND Single Nucleotide Polymorphisms AND Enamel formation genes |
| Google Scholar & Opengrey | Dental caries AND Single nucleotide Polymorphisms OR genetic polymorphisms AND genes OR Genetic Loci AND genome wide association Scan |

Supplementary Table 2 - Table of characteristics of Included studies

| S. No. | Gene/Single-nucleotide Polymorphism (SNP) | Chromosome / Functional consequence | Author(s)/year | Study Design/Country/Age | Sample Size | Genotype Frequencies - Cases vs. Controls | Allele Frequencies - Cases vs. Controls | Statistical Test(s) Done | *P* value | Results |
| --- | --- | --- | --- | --- | --- | --- | --- | --- | --- | --- |
| 1 | LTF / rs1126478 | 3p21.31/ Coding Sequence; missense variant | Wang et al., 2017 [25] | Case control / China / under 4 years | 1005; Caries free - 500; Severe Caries - 505 | GG/AG/AA - 219 / 222/ 64 Vs 227/209 / 64 | G/A - 660 / 350 Vs 663 /337 | MVA - OR 95% CI - Genotype -AG -β = 0.106; OR =1.112 (0.724 - 1.708); Genotype AA -β = 0.062; OR =1.604 (0.794 - 1.425); Chi Square Test - Genotype -AG -χ2- 0.507 (compared to GG); AA -χ2 = 0.032 (compared to GG); Allele— UVA - G/A -χ2= 0.203; | Genotype AG - 0.476; AA - 0.858; Allele - UVA - 0.652 | No difference between severe caries and caries free children |
|  |  |  | Wang et al., 2018 [29] | Case control / China / 24 to 48 months | 910; 403 - no caries or white-spot lesions; 230 - moderate caries (8 ≤ dmft ≤ 12); 277 with severe caries (13 ≤ dmft ≤ 20) | AA / GA/ GG; Caries free -- 51 /168 /184 Mod.Caries- 28 / 95 / 107 Sev. Caries = 35 /129 / 113 | A/G. Caries free -270 / 536 Mod.Caries - 151 / 309 Sev. Caries --199 / 355; Haplotype G - A = 267.43 Vs 350.00; Haplotype G - G =195.57 Vs 242.00 | Chi Square - Genotypes Caries free Vs moderate Caries -χ2 = 0.06; Moderate caries Vs severe Caries --χ2 = 1.76; Caries Free Vs Severe Caries - χ2 = 1.79 ; Alleles -Caries free Vs moderate Caries - χ2 ^=^  0.06; Moderate caries Vs severe Caries - χ^2^ - 1.06; Caries Free Vs Severe Caries - χ2^-^ 0.85; Haplotype G - A - χ2 = 0.30; OR (95% CI) = 1.06 [0.87-1.28] Haplotype G - G - χ2= 0.06; OR(95% CI) = 0.97 [0.79-1.21] | Genotypes - Caries free Vs moderate Caries - 0.97; Moderate caries Vs severe Caries - 0.41; Caries Free Vs Severe Caries - 0.41; Alleles - Caries free Vs moderate Caries - 0.81; Moderate caries Vs severe Caries - 0.30; Caries Free Vs Severe Caries - 0.36 |  |
|  |  |  | Wu et al., 2020 [32] | Case Control / Chinese / 3 - 5 years. | 517; No Caries 265; With caries 254 (Mild caries 85, Moderate caries 83, Severe caries 86) | Not Mentioned | Not mentioned | Chi Square - χ2 Mild caries- 0.429; Mod. caries-4.621; Severe caries - 0.319; Mild + Mod Caries - 2.500; Mod + Severe caries - 2.321; Mild + Mod + severe caries - 1.769; **OR**  Mild caries- 1.189; Mod.Caries-1.767; Severe caries - 1.161; Mild + Mod.Caries - 1.442; Mod + Severe caries - 1.423; Mild + Mod + severe caries - 1.340;  **OR Additive model**  Mild caries-1.179; Mod.Caries-1.809; Severe caries - 1.155; Mild + Mod.Caries - 1.443; Mod + Severe caries - 1.425; Mild + Mod + severe caries - 1.336; **OR Dominant model**  Mild caries-1.287; Mod.Caries-2.468; Severe caries - 1.235; Mild + Mod.Caries - 1.722; Mod + Severe caries - 1.678; Mild + Mod + severe caries - 1.529  **OR Recessive model**  Mild caries-1.218; Mod.Caries-1.943; Severe caries - 1.198; Mild + Mod.Caries - 1.554; Mod + Severe caries - 1.540; Mild + Mod + severe caries - 1.428; | Mild caries- 0.513; Mod. caries -0.032; Severe caries - 0.572; Mild + Mod Caries - 0.114; Mod + Severe caries - 0.128; Mild + Mod + severe caries - 0.184; **Additive model**  Mild caries-0.591; Mod.Caries-0.051; Severe caries - 0.637; Mild + Mod.Caries - 0.170; Mod + Severe caries- 0.185; Mild + Mod + severe caries - 0.255; **Dominant model**  Mild caries- 0.492; Mod.Caries-0.055; Severe caries - 0.563; Mild + Mod.Caries-0.097; Mod + Severe caries-0.112; Mild +Mod+severe caries - 0.164; Recessive Model Mild caries-0.730; Mod. Caries-0.222; Severe caries -0.753; Mild+Mod.Caries -0.376; Mod + Severe caries -0.386; Mild+Mod+ severe caries- 0.456; | No association |
| 2 | LTF / rs1126477 | 3p21.31/ Coding sequence; missense variant; 5’ UTR variant | Wang et al., 2018 [29] | Case Control /China / 24 to 48 months | 910; 403 - no caries or white-spot lesions; 230 - moderate caries (8 ≤ dmft ≤ 12); 277 with severe caries (13 ≤ dmft ≤ 20). | GG /AG / AA; Caries free - 138 /187 / 78 Mod.Caries -- 76 /112 / 42 Sev. Caries -99 / 130 /48 | G/ A Caries free --463 / 343 Mod.Caries --264 / 196 Sev. Caries --328 / 226; Haplotype A - A = 2.57 Vs 0.00 Haplotype A - G = 340.43 Vs 422.00 | Chi Square - Genotypes Caries free Vs moderate Caries -χ2 = 0.32; Moderate caries Vs severe Caries --χ2 = 0.41; Caries Free Vs Severe Caries - χ2 - 0.48; Alleles - Caries free Vs moderate Caries —χ2 <0.01; Moderate caries Vs severe Caries — χ2 - 0.34; Caries Free Vs Severe Caries - χ2 - 0.42; Haplotype A -A = NA Haplotype Analysis A - G - χ2= 0.11; OR (95% CI) = 0.97 [0.80-1.17] | Chi Square - Genotypes Caries free Vs moderate Caries - 0.99 Moderate caries Vs severe Caries - 0.82 Caries Free Vs Severe Caries - 0.52 Alleles - Caries free Vs moderate Caries - 0.85 Moderate caries Vs severe Caries - 0.56; Caries Free Vs Severe Caries - 0.79 | No association between SNP and severity of caries |
| 3 | LTF / rs2269436 | 3p21.31/ Intron variant | Abbasoğlu et al., 2015 [18] | Cross Sectional / Turkey / 2-5 years | 259; Caries free - 123; Caries experience - 136 | AA/AG/GG - Not mentioned | A/G - Not mentioned | UniVariate Analysis - OR (95%CI ) ref - AG - 1.12 (0.50–2.50); GG - 2.68 (0.27–26.2); MultiVariate Analysis -OR (95%CI ) ref - AG - 1.34 (0.55–3.26); GG - 1.77 (0.18–17.5); | UniVariate Analysis ref - AG - 0.787 GG - 0.396 MultiVariate Analysis ref - AG - 0.521 GG - 0.627 | No association |
| 4 | LTF / rs743658 | 3p21.31/ Intron variant | Abbasoğlu et al., 2015 [18] | Cross Sectional / Turkey / 2-5 years | 259; Caries free - 123; Caries experience - 136 | AA/AG/GG - Not mentioned | A/G - Not mentioned | UniVariate Analysis -OR (95%CI ) AA - AG -0.38 (0.03–4.24); GG -0.37 (0.03–3.69); MultiVariate Analysis -OR (95%CI ) AA - AG - 0.69 (0.06–7.8); GG - 0.59 (0.06–5.89); | UniVariate Analysis AA - AG - 0.438 GG - 0.403 MultiVariate Analysis AA - AG - 0.769 GG - 0.657 | No association |
| 5 | LTF / rs4547741 | 3p21.31/ Intron variant | Abbasoğlu et al., 2015 [18] | Cross Sectional / Turkey / 2-5 years | 259; Caries free - 123; Caries experience - 136 | AA/AG/GG - Not mentioned | A/G; Not mentioned | UniVariate Analysis -OR (95%CI ) CC - CT -0.47 (0.23–0.95); TT -0.38 (0.03–4.21); MultiVariate Analysis -OR (95%CI ) CC - CT - 0.44 (0.21–0.96) TT - 0.24 (0.02–2.79) | UniVariate Analysis CC - CT - 0.036 TT - 0.427 MultiVariate Analysis CC - CT - 0.038 TT - 0.257 | Genotype CT protective for ECC |
|  |  | 3p21.31 | Al-Marshad et al / 2021 [30] | Case-Control / Saudi-Arabia/2 - 6 yrs | 360; No Caries - 98; With Caries - 262 | CC/CT/TT _ Not mentioned | C/T - Not mentioned | Univariate analysis - OR (95%CI ) CC - CT -0.96 (0.493 - 1.868) TT - 0.242 (0.04 - 1.475)) Multivariate analysis - OR (95%CI ) - CC - CT - 0.669 (0.314 - 1.434) TT - 0.283 (0.039 - 2.048) | Univariate analysis CT - 0.904; TT - 0.124 Multivariate analysis CT - 0.301; TT - 0.211 | No association |
|  |  |  | Wu et al., 2020 [32] | Case Control / Chinese / 3 - 5 years. | 517; No Caries 265; With caries 254 (Mild caries 85, Moderate caries 83, Severe caries 86) | Not Mentioned | Not mentioned | Chi Square - χ2 Mild caries- 0.446; Mod. caries-0.116; Severe caries - 0.302; Mild + Mod Caries - 0.030; Mod + Severe caries - 0.014; Mild + Mod + severe caries - 0.122; **OR**  Mild caries- 0.731; Mod.Caries-1.159; Severe caries - 0.778; Mild + Mod.Caries -0.935; Mod + Severe caries - 0.956; Mild + Mod + severe caries - 0.880;  **OR Additive model**  Mild caries-NA; Mod.Caries-NA; Severe caries - NA; Mild + Mod.Caries - NA; Mod + Severe caries - NA; Mild + Mod + severe caries - NA; **OR Dominant model**  Mild caries- 0.708; Mod.Caries-1.182; Severe caries - 0.758; Mild + Mod.Caries - 0.928; Mod + Severe caries - 0.950; Mild + Mod + severe caries - 0.867  **OR Recessive model**  Mild caries-NA; Mod.Caries-NA; Severe caries - NA; Mild + Mod.Caries - NA; Mod + Severe caries - NA; Mild + Mod + severe caries - NA; | Mild caries- 0.504; Mod. caries-0.733; Severe caries - 0.583; Mild + Mod Caries - 0.863; Mod + Severe caries - 0.906; Mild + Mod + severe caries - 0.727; **Additive model**  Mild caries-NA; Mod.Caries-NA; Severe caries - NA; Mild + Mod.Caries - NA; Mod + Severe caries - NA; Mild + Mod + severe caries - NA; **Dominant model**  Mild caries- 0.484; Mod.Caries-0.717; Severe caries - 0.564; Mild + Mod.Caries - 0.855; Mod + Severe caries - 0.901; Mild + Mod + severe caries - 0.713; Recessive Model Mild caries-NA; Mod.Caries-NA; Severe caries - NA; Mild + Mod.Caries - NA; Mod + Severe caries - NA; Mild + Mod + severe caries - NA; | No association |
|  |  | 3p21.31 | Zaorska et al / 2021 [31] | Case-Control / Poland/ 2 - 3 years | 95; No caries - 47; With caries - 48 | CC/CT/TT - 35/12/1 Vs 32/14/1 | C/T = 82/14 Vs 78/16 | OR (95% CI); CC Vs CT +TT = 1.3 (0.5 to 3.1); CT Vs CC+TT = 0.8 (0.3 to 1.9); TT Vs CC + CT = 1.0 (0.1 to 16.1); Alleles T Vs C = 0.8 (0.4 to 1.8) | Genotype P Value - CC Vs CT + TT = 0.6059; CT Vs CC +TT = 0.6012; TT Vs CC + CT = 0.9880; Alleles P value = 0.6452 |  |
| 6 | LTF / rs17078878 | 3p21.31/ Intron variant | Abbasoğlu et al., 2015 [18] | Cross Sectional / Turkey / 2-5 years | 259; Caries free - 123; Caries experience - 136 | AA/AG/GG - Not mentioned | A/G; Not mentioned | UniVariate Analysis -OR (95%CI ) AA - AC -0.35 (0.03–3.67); CC -0.41 (0.04–3.99); MultiVariate Analysis -OR (95%CI ) AA - AC -0.60 (0.06–6.53); CC - 0.61 (0.06–6.01); | UniVariate Analysis -OR (95%CI ) AA - AC - 0.382 CC - 0.441 MultiVariate Analysis -OR (95%CI ) AA - AC - 0.676 CC - 0.669 | No association |
| 14 | DEFB1 / rs11362 | 8p23.1/ 5’ UTR variant | Mubayrik et al 2014 [28] | Case Control / Turkey / 3 - 6 years | 178; No caries - 82; With dental caries - 96 | AA/GA/ GG - Cases Vs Controls = 17/38/37 Vs 12/41/33 | Not given | OR (95% CI)- 0.93 (0.51-1.69); | p - 0.61 | No association |
|  |  |  | Abbasoğlu et al., 2015 [18] | Cross Sectional / Turkey / 2-5 years | 259; Caries free - 123; Caries experience - 136 | CC/CT/TT - Not mentioned | C/T - Not mentioned | UniVariate Analysis -OR (95%CI ) CC - CT -0.98 (0.56–1.74). TT -0.97 (0.49–1.92); MultiVariate Analysis -OR (95%CI ) CC - CT -0.93 (0.51–1.71); TT - 0.85 (0.41–1.77); | UniVariate Analysis - CC - CT - 0.98 TT - 0.97 MultiVariate Analysis CC - CT - 0.818 TT - 0.667 | No association |
|  |  |  | Lips et al., 2017 [35] | Cohort / Brazil / 2-6 years | 510; Caries free- 342; Caries Experience - 168 | TT/TC/CC - 20/62/70 Vs 43/133 /128 | T/C = 102/ 202 Vs 219 / 389 | Chi Square test done - Value not given. Genotype - 0.712; Alleles -p=0.462 | Genotype - 0.712; Alleles - 0.462 | No association |
|  |  |  | Weber et al., 2018 [36] | Cohort / Norway at 5 years | 876; No Caries - 647; With Caries (low caries + high caries) - 228 | CC/CT/TT Frequencies not given | C/T - Cases Vs Controls No primary Caries Vs High Primary Caries - Recessive = 19/59 Vs 74/410 | Chi Square - Alleles - 4.001; | p - 0.004 | No association |
|  |  |  | Wu et al., 2020 [32] | Case Control / Chinese / 3 - 5 years. | 517; No Caries 265; With caries 254 (Mild caries 85, Moderate caries 83, Severe caries 86) | Not Mentioned | Not mentioned | Chi Square - χ2 Mild caries- 6.682; Mod. Caries-7.962; Severe caries -18.850; Mild + Mod Caries - 9.259; Mod + Severe caries - 16.500; Mild + Mod + severe caries - 14.850; **OR —** Mild caries - 2.040 (1.819-2.485); Mod.Caries- 2.192 (1.999-2.308); Severe caries-3.234 (3.039-3.506); Mild + Mod.Caries -2.112 (2.081-2.427); Mod + Severe caries - 2.683 (2.151-2.947); Mild + Mod + severe caries-2.447 (2.275-2.704);  **OR Additive model**  Mildcaries- 1.946 (1.360 - 2.238); Mod.Caries- 2.519 (1.145-3.941); Severe caries -3.484 (1.897 - 5.359); Mild + Mod.Caries-2.275 (1.538-2.857); Mod + Severe caries- 2.915 (1.124-4.722); Mild + Mod + severe caries -2.625 (1.297-3.359); **OR Dominant model**  Mild caries- 2.801 (1.292- 4.806); Mod.Caries- 2.933 (1.441-5.619); Severe caries-4.444 (2.761-8.639); Mild+ Mod. Caries- 2.331(1.734-4.581); Mod + Severe caries-2.877 (1.763-4.615); Mild + Mod + severe caries-2.849 (1.394-4.628)  **OR Recessive model**  Mild caries-2.252 (1.170-6.988); Mod.Caries- 5.556(2.842-14.695); Severe caries- 6.098 (4.054-18.794); Mild + Mod.Caries-3.729(1.558 - 8.785); Mod + Severe caries-5.816(1.580-18.440); Mild + Mod + severe caries- 4.484 (2.386-12.398) | Mild caries-9.74E-03; Mod. caries -4.78E-03; Severe caries-1.42E-05; Mild + Mod Caries -2.34E-03; Mod + Severe caries -4.87E-05; Mild+ Mod+severe caries-1.17E-04; **Additive model**  Mild caries-0.074; Mod.Caries-7.284E-03; Severe caries-3.802E-04; Mild + Mod.Caries-0.012; Mod + Severe caries-9.739E-04; Mild + Mod + severe caries-2.487E-03; **Dominant model**  Mild caries - 5.493E-03; Mod.Caries-0.0372; Severe caries-1.446E-04; Mild + Mod.Caries -7.990E-03; Mod + Severe caries-1.064E-03; Mild + Mod + severe caries-5.435E-04; Recessive Model Mild caries-0.255; Mod.Caries-0.010; Severe caries - 6.109E-03; Mild + Mod.Caries - 0.039; Mod + Severe caries - 5.055E-03; Mild + Mod + severe caries - 0.016; | No association |
| 15 | DEFB1 / rs1799946 | 8p23.1/ 5’ UTR variant | Lips et al.,2017 [35] | Cohort / Brazil / 2-6 years | 510; Caries free- 342; Caries Experience - 168 | TT/TC/CC - cases = 41/73/41 Vs 64/160 /92 | T/C -155 / 155 Vs 288 / 344 | Chi Square test done - Value not given. | Genotype - 0.314; Alleles - 0.200 | No association |
|  |  |  | Wu et al., 2020 [32] | Case Control / Chinese / 3 - 5 years. | 517; No Caries 265; With caries 254 (Mild caries 85, Moderate caries 83, Severe caries 86) | Not Mentioned | Not mentioned | Chi Square - χ2 Mild caries- 0.353; Mod. Caries-0.573; Severe caries -8.424; Mild + Mod Caries-0.598; Mod + Severe caries-4.544; Mild + Mod + severe caries-2.713; **OR** Mild caries - 0.861 (0.719-1.022); Mod.Caries- 0.824 (0.632-1.106); Severe caries-0.473 (0.347-0.789); Mild + Mod.Caries -0.843 (0.735 -1.020); Mod + Severe caries-0.623 (0.359- 0.838); Mild + Mod + severe caries- 0.738(0.581-0.927);  **OR Additive model**  Mildcaries-0.873(0.718-1.134); Mod.Caries-0.853 (0.716- 1.020); Severe caries -0.984 (0.831-1.173); Mild + Mod.Caries-0.863 (0.705-1.077); Mod + Severe caries- 0.659 (0.369-0.810); Mild + Mod + severe caries -0.726 (0.581- 0.929); **OR Dominant model**  Mild caries- 0.730(0.618- 0.820); Mod.Caries- 0.606 (0.310-0.836); Severe caries-0.387 (0.255-0.579); Mild+ Mod. Caries- 0.667 (0.405-0.906); Mod + Severe caries-0.478 (0.267-0.611); Mild + Mod + severe caries-0.550 (0.406-0.904)  **OR Recessive model**  Mild caries- 0.941(0.725- 1.186); Mod.Caries-1.043 (0.886 -1.396); Severe caries- 0.350(0.210-0.536); Mild + Mod.Caries-0.990 (0.719-1.222); Mod + Severe caries-0.651 (0.409-0.804); Mild + Mod + severe caries- 0.745 (0.544-0.944) | Mild caries- 0.552; Mod. caries -0.449; Severe caries-3.70E-03; Mild + Mod Caries -0.440; Mod + Severe caries - 0.033; Mild+ Mod+severe caries-0.147; **Additive model**  Mild caries-0.074; Mod.Caries-7.284E-03; Severe caries-3.802E-04; Mild + Mod.Caries-0.012; Mod + Severe caries-9.739E-04; Mild + Mod + severe caries-2.487E-03; **Dominant model**  Mild caries - 0.429; Mod.Caries-0.210; Severe caries- 0.014; Mild + Mod.Caries -0.249; Mod + Severe caries-0.034; Mild + Mod + severe caries-0.072; Recessive Model Mild caries-0.883; Mod.Caries-0.919; Severe caries - 0.035; Mild + Mod.Caries - 0.977; Mod + Severe caries - 0.254; Mild + Mod + severe caries - 0.398; | No association |
| 16 | DEFB1 / rs1800972 | 8p23.1/ 5’ UTR variant | Mubayrik et al 2014 [28] | Case Control / Turkey / 3 - 6 years | 178; No caries - 82; With dental caries - 96 | CC/CG/GG - Cases Vs Controls = 67/24/2 Vs 61/19/3 | Not given | OR (95% CI) - 1.08 (0.55-2.01). | Genotype - 0.61 | No association |
|  |  |  | Abbasoğlu et al., 2015 [18] | Cross Sectional / Turkey / 2-5 years | 259; Caries free - 123; Caries experience - 136 | CC/CG/GG - Frequencies not mentioned | C/G - Frequencies not mentioned | UniVariate Analysis -OR (95%CI ) CC - CG -1.89 (0.39–9.22); GG -1.35 (0.29–6.18); MultiVariate Analysis -OR (95%CI ) CC - CG -2.57 (0.45–14.7); GG -1.39 (0.26–7.34); | UniVariate Analysis - CC - CG - 0.432 GG - 0.701 MultiVariate Analysis -OR (95%CI ) CC - CG - 0.287 GG - 0.700 | No association |
|  |  |  | Weber et al., 2018 [36] | Cohort / Norway at 5 years | 876; No Caries - 647; With Caries (low caries + high caries) - 228 | CC/CG/GG - Cases Vs Controls Frequencies not given | C/G- Cases Vs Controls; No primary Caries Vs High Primary Caries -Recessive = 4/ 45 Vs 6 /288 | Plink Software - Genotypic (2df) test; Chi Square - Allele = 5.562. | p=0.02 | No association |
| 17 | ALOX15 / rs2619112. | 17p13.2 / Intron Variant | Abbasoğlu et al., 2015 [18] | Cross Sectional / Turkey / 2-5 years | 259; Caries free - 123; Caries experience - 136 | CC/AG/GG - Not mentioned | Not mentioned | UniVariate Analysis -OR (95%CI ) CC - - AG -0.95 (0.50–1.79); GG -0.79 (0.39–1.61) MultiVariate Analysis -OR (95%CI ) CC - AG -1.06 (0.54–2.09); GG - 0.95 (0.44–2.02); | UniVariate Analysis - CC - - AG - 0.864 GG - 0.521 MultiVariate Analysis CC - AG - 0.869 GG - 0.886 | No association |
|  |  | 17p13.2 | Weber et al., 2018 [36] | Cohort / Norway at 5 years | 876; No Caries - 647; With Caries (low caries + high caries) - 228 | AA/AG/GG - No primary Caries Vs Low primary Caries - 14/67/30 Vs 100/278/211 | A/G - Frequencies not given | Plink Software - Genotypic (2df) test; Chi Square - Allele = 5.562. | p - 0,04 | No association |
| 18 | ALOX15 / rs7217186 | 17p13.2 / Intron Variant | Abbasoğlu et al., 2015 [18] | Cross Sectional / Turkey / 2-5 years | 259; Caries free - 123; Caries experience - 136 | CC/CT/TT - Frequencies not mentioned | C/T - Frequencies Not mentioned | UniVariate Analysis -OR (95%CI ) CC - - CT -0.63 (0.23–1.72); TT - 2.57 (0.96 - 6.92); MultiVariate Analysis -OR (95%CI ) CC - CT - 0.52 (0.18–1.54); TT - 2.97 (1.00–8.86); | UniVariate Analysis - CC - - CT - 0.368 TT - 0.061 MultiVariate Analysis CC - CT - 0.239 TT - 0.050 | Genotype TT risk factor for ECC |
|  |  | 17p13.2 | Weber et al., 2018 [36] | Cohort / Norway at 5 years | 876; No Caries - 647; With Caries (low caries + high caries) - 228 | CC/CT/TT - Frequencies not mentioned | C/T - Frequencies Not mentioned | Student’s t test - mean differences; χ2 or Fisher’s exact tests - difference in frequencies between caries-free & caries experience; Logistic regression analysis - genetic marker. Plink Software - genotypic (2df) Test used. | VALUES NOT GIVEN | No association |
|  |  | 17p13.2 | Zaorska et al / 2021 [31] | Case-Control / Poland/ 2 - 3 years | 95; No caries - 47; With caries - 48 | CC/CT/TT - 13/23/12 Vs 13/21/13 | C/T = 49/47 Vs 47/47 | OR (95% CI); CC Vs CT +TT = 1.0 (0.4 to 2.4); CT Vs CC+TT = 1.1 (0.5 to 2.6); TT Vs CC + CT = 0.9 (0.4 to 2.2); Alleles T Vs C = 1.0 (0.5 to 1.7) | Genotype P Value - CC Vs CT + TT = 0.9498; CT Vs CC +TT = 0.7519; TT Vs CC + CT = 0.7686; Alleles P value = 0.8858 |  |
| 19 | MBL2 / rs1800450 | 10q21.1 / Coding Sequence, Missense variant | Olszowski et al., 2012 [26] | Case control / Poland /5 years | 71; Low caries exp.=34 & High caries Exp. =37 | GG/AG/AA = 28 / 9 /0 Vs 30/4/ 0 | G/A = 65 /9 Vs 64 /4; Carriage of Allele A = 9 /4 | Odds Ratio - 95% CI Allele -G/A = 2.22 (0.65 - 7.56); Fisher’s Exact Test - Comparison with wild type homozygote - C/G = OR - Carriage of Allele A = 2.41 (0.67–8.72). | Odds Ratio - 95% CI Allele -G/A - 0.25; Carriage of Allele A - 0.23 | No significant difference between cases and controls. But may be a risk factor when analysed as Haplotype |
| 20 | MBL2 / rs7096206; | 10q21.1 / Genic, Upstream Transcript variant; Intron Variant; UTV | Olszowski et al., 2012 [26] | Case control / Poland /5 years | 71; Low caries exp .=34 & High caries Exp. =37 | CC/CG/GG = 21/15 /1 Vs 28/5/1 | C/G = 57/17 Vs 61/7; Carriage of Allele G = 16 /6 | Odds Ratio 95% CI Allele Frequency- 2.60 (1.00 - 6.73); Fisher’s exact test for comparison with wild-type homozygote - G/A = OR - Carriage of Allele G = 3.56 (1.19–10.64). | Odds Ratio 95% CI Allele Frequency - 0.071; Carriage of Allele G - 0.23 | G allele more in children with caries |
|  |  | 10q21.1 | Zaorska et al / 2021 [31] | Case-Control / Poland/ 2 - 3 years | 95; No caries - 47; With caries - 48 | CC/CG/ GG - 9/21/18 Vs 6/21/20 | C/G = 78/18 Vs 62/32 | OR (95% CI); CC Vs CG +GG = 1.9 (0.8 to 4.3); CG Vs GG+TT = 1.0 (0.4 to 2.3); GG Vs CG + GT = 0.1 (0.01 to 0.9); Alleles G Vs C = Not mentioned | Genotype P Value - CC Vs CG + GG = 0.1254; CG Vs CC + GG = 0.9417; GG Vs CC + CG = 0.0363; Alleles P value = 0.0180 | Not associated with caries |
|  | MBL2 / rs11003125 | 10q21.1/  Genic upstream transcript variant, intron_variant | Wu et al., 2020 [32] | Case Control / Chinese / 3 - 5 years. | 517; No Caries 265; With caries 254 (Mild caries 85, Moderate caries 83, Severe caries 86) | Not Mentioned | Not mentioned | Chi Square - χ2 Mild caries- 0.122; Mod. caries-0.061; Severe caries - 0.227; Mild + Mod Caries - 0.118; Mod + Severe caries - 0.175; Mild + Mod + severe caries- 0.190; **OR**  Mild caries- 0.916; Mod.Caries-0.939; Severe caries - 0.886; Mild + Mod.Caries -0.927; Mod + Severe caries -0.911; Mild + Mod + severe caries - 0.913;  **OR Additive model**  Mild caries- 0.928; Mod.Caries-0.948; Severe caries - 0.905; Mild + Mod.Caries - 0.937; Mod + Severe caries - 0.925; Mild + Mod + severe caries - 0.926; **OR Dominant model**  Mild caries- 0.618; Mod.Caries-0.662; Severe caries - 0.563; Mild + Mod.Caries - 0.639; Mod + Severe caries - 0.608; Mild + Mod + severe caries - 0.612  **OR Recessive model**  Mild caries- 1.310 Mod.Caries-1.299; Severe caries -1.336; Mild + Mod. Caries -1.305; Mod + Severe caries-1.318; Mild + Mod + severe caries - 1.315 | Mild caries- 0.727; Mod. caries-0.804; Severe caries - 0.634; Mild + Mod Caries - 0.731; Mod + Severe caries - 0.675; Mild + Mod + severe caries - 0.663; **Additive model**  Mild caries-0.783; Mod. Caries- 0.846; Severe caries - 0.710; Mild+Mod.Caries - 0.791; Mod+Severe caries-0.747; Mild+Mod+severe caries - 0.739; **Dominant model**  Mild caries- 0.255; Mod.Caries-0.337; Severe caries - 0.171; Mild + Mod.Caries - 0.236; Mod + Severe caries - 0.187; Mild + Mod + severe caries - 0.172; Recessive Model Mild caries-0.543; Mod.Caries-0.562;Severe caries - 0.515; Mild + Mod.Caries-0.500Mod + Severe caries - 0.484; Mild+Mod+severe caries -0.465; | No association |
| 21 | MBL codons | 10q21.1 | Yang et al., 2013 [27] | Case control / China / 1-5 years | 130; No caries - 68; Severe Caries - 62 | Codon 54 - GGC/GGC - 38 Vs 51; GGC/GAC - 22vs16; GAC/GAC - 2 vs 1 GG/GA/AA = 38 /22 / 2 Vs 51 /16 /1 | Allele Frequencies- Wild Allele -“A” Allele- GGC/GGC- 98 Vs 118; Mutant allele “B” Allele-GGC/GAC-22 Vs16 G/A = 98/22 Vs118 /16 | Chi-Square test - Codon 54 Genotype Frequencies - GGC/GGC Vs GGC/GAC Vs GAC/GAC =χ2 = 2.82; Allele Frequencies - GGC/GGC Vs GGC/GAC -χ2= 2.76; | Chi-Square test - Codon 54 Genotype Frequencies - 0.093 Allele Frequencies - GGC/GGC Vs GGC/GAC - 0.097 | mutant genotype (GGC/GAC and GAC/GAC) was more frequent among children with S-ECC but did not significantly differ |
| 22 | MASP2 /rs72550870 | 1p36.22 / Missense variant, Coding Sequence variant, non coding Transcript variant | Olszowski et al., 2012 [26] | Case control / Poland /5 years | 71; Low caries exp.=34 & High caries Exp. =37 | AA/AG/GG = 32/5/0 Vs 31/3/0 | A/G =106 /10 Vs 96 /4 - Carriage of Allele G =5 / 3 | Odds Ratio 95% CI - Allele - 1.57 (0.36–6.83); Fisher’s exact test for comparison with wild-type homozygote - Carriage of Allele G =1.61 (0.36–7.34); | Odds Ratio 95% CI - Allele - 0.72; Carriage of Allele G - 0.71 | No association with caries |
| 23 | TNF-alpha / rs1800629 | 6p21.33/2 KB upstream variant; UTV | Wang et al., 2017 [25] | Case control / China / under 4 years | 1005; Caries free - 500; Severe Caries - 505 | GG/AG/AA - 459 / 44 / 2 Vs 433/ 66 /1 | G/A -962 /48 Vs 932/68 | MVA - OR 95% CI - Genotype - AG -β = 0.676; OR = 0.526 (0.338 - 0.820); Genotype AA - β = 0.062; OR =1.966 (0.114 - 33.946) - Allele - chiSquare = 3.847; Chi Square Test - Genotype - A/G - -χ2- 5.142; UVA- Allele G/A -χ2= 0.203 | Allele - 0.049; Genotype - 0.023 | AG genotype protective for caries as compared to GG genotype. |
| 24 | LPO / rs8178350 | 17q22 / Intron variant | Stanley et al., 2014 [34] | Cohort / US / IHS - 3 to 5 years; IFS - 4 to 6 years | 200 [IHS - 64 (41 whites + 23 blacks) + IFS 136] | Not mentioned | Not mentioned | Linear and Logistic regression. | IHS (Whites) - 0.7573; IHS (Blacks) - 0.0238; IFS (Whites) - 0.127 | No association |
| 25 | LPO / rs7209537 | 17q22 / UTV, Intron variant, Genic UTV | Stanley et al., 2014 [34] | Cohort / US / IHS - 3 to 5 years; IFS - 4 to 6 years | 200 [IHS - 64 (41 whites + 23 blacks) + IFS 136] | Not mentioned | Not mentioned | Linear and Logistic regression. | IHS (Whites) - 0.7342; IHS (Blacks) - 0.3868; IFS (Whites) - 0.0407 | No association |
| 26 | LPO / rs17762644 | 17q22 / Intron Variant | Stanley et al., 2014 [34] | Cohort / US / IHS - 3 to 5 years; IFS - 4 to 6 years | 200 [IHS - 64 (41 whites + 23 blacks) + IFS 136] | Not mentioned | Not mentioned | Linear and Logistic regression. | IHS (Whites) - 0.9853; IHS (Blacks) - 0.5215; IFS (Whites) - 0.7733 | No association |
| 27 | LPO / rs8178281 | 17q22/ Intron Variant, Genic UTV | Stanley et al., 2014 [34] | Cohort / US / IHS - 3 to 5 years; IFS - 4 to 6 years | 200 [IHS - 64 (41 whites + 23 blacks) + IFS 136] | Not mentioned | Not mentioned | Linear and Logistic regression. | IHS (Whites) - 0.5921; IHS (Blacks) - NA; IFS (Whites) - 0.2874 | No association |
| 28 | LPO / rs8178290 | 17q22/ Intron Variant, Genic UTV | Stanley et al., 2014 [34] | Cohort / US / IHS - 3 to 5 years; IFS - 4 to 6 years | 200 [IHS - 64 (41 whites + 23 blacks) + IFS 136] | Not mentioned | Not mentioned | Linear and Logistic regression | IHS (Whites) - 0.6098; IHS (Blacks) - 0.5518; IFS (Whites) - 0.5146 | No association |
| 29 | LPO / rs8178307 | 17q22/ Intron Variant, Genic UTV | Stanley et al., 2014 [34] | Cohort / US / IHS - 3 to 5 years; IFS - 4 to 6 years | 200 [IHS - 64 (41 whites + 23 blacks) + IFS 136] | Not mentioned | Not mentioned | Linear and Logistic regression | IHS (Whites) - 0.6385; IHS (Blacks) - 0.5518; IFS (Whites) - 0.5049 | No association |
| 30 | LPO / rs8178329 | 17q22/ synonymous variant,genic upstream transcript variant, non-coding_ transcript_ variant,coding_sequence_variant | Stanley et al., 2014 [34] | Cohort / US / IHS - 3 to 5 years; IFS - 4 to 6 years | 200 [IHS - 64 (41 whites + 23 blacks) + IFS 136] | Not mentioned | Not mentioned | Linear and Logistic regression | IHS (Whites) - 0.9782; IHS (Blacks) - 0.1183; IFS (Whites) - 0.385 | No association |
| 31 | LPO / rs3744093 | 17q22 / Intron variant; Genic UTV, Coding Sequence variant; Missense variant | Stanley et al., 2014 [34] | Cohort / US / IHS - 3 to 5 years; IFS - 4 to 6 years | 200 [IHS - 64 (41 whites + 23 blacks) + IFS 136] | Not mentioned | Not mentioned | Linear and Logistic regression | IHS (Whites) - 0.4486; IHS (Blacks) - 0.4833; IFS (Whites) - 0.1102 | No association |
|  | TRAV4 / rs1997532 | 14q11.2 | Briseño-Ruiz et al., 2013 [33] | Case Control / Turkey / 3 to 5 years | 172 - Caries free = 82; Caries experience (dmft > 4) = 90 | CC/CT/TT - 20/59/26 Vs 8/41/37 | C/T - 79 /111 Vs 57/115 | Chi-square, Fisher’s exact and logistic regression. Allele (Model free) - OR (95% CI) = 0.7 (0.45 -1.07) Recessive Model - OR (95% CI) = 0.44 (0.24 - 0.81) | Genotype - p = 0.01; Allele (Model free) - p = 0.01; Recessive Model - p =0.007; | T allele associated with low caries experience |
|  |  |  | Weber et al., 2018 [36] | Cohort / Norway / 5 years | 876; No Caries - 647; With Caries (low caries + high caries) - 228 | Not mentioned | Not mentioned | Plink Software - genotypic (2df) Test; Chi-square test | Not mentioned | No Association |
|  | TRAV4 / rs8011979 | 14q11.2 | Briseño-Ruiz et al., 2013 [33] | Case Control / Turkey / 3 to 5 years | 172 - Caries free = 82; Caries experience (dmft > 4) = 90 | CC/CT/TT - 30/51/21 Vs 39/37/5 | C/T -111/93 Vs 115/47 | Chi-square, Fisher’s exact and logistic regression. Allele (Model free) - OR (95% CI) = 0.49 (0.31 -0.75) Recessive Model -OR (95% CI) = 0.45 (0.24 - 0.82) | Genotype - p = 0.04; Allele (Model free) - p = 0.01; Recessive Model - p =0.009; | T allele associated with low caries experience |
|  |  |  | Weber et al., 2018 [36] | Cohort / Norway / 5 years | 876; No Caries - 647; With Caries (low caries + high caries) - 228 | Not mentioned | Not mentioned | Plink Software - genotypic (2df) Test; chi-square test | Not mentioned | No Association |
|  | TRAV4 / rs7150049 | 14q11.2 | Briseño-Ruiz et al., 2013 [33] | Case Control / Turkey / 3 to 5 years | 172 - Caries free = 82; Caries experience (dmft > 4) = 90 | AA/AG/GG - 29/51/22 Vs 38/40/5 | A/G - 109/95 Vs 116/50 | Chi-square, Fisher’s exact and logistic regression . Allele (Model free) - OR (95% CI) = 0.49 (0.32 -0.76) Recessive Model - OR (95% CI) = 0.47 (0.26 - 0.95) | Genotype - p = 0.03; Allele (Model free) - p = 0.01; Recessive Model - p =0.001; | G allele associated with low caries experience |
|  |  |  | Weber et al., 2018 [36] | Cohort / Norway / 5 years | 876; No Caries - 647; With Caries (low caries + high caries) - 228 | Not mentioned | Not mentioned | Plink Software - genotypic (2df) Test; Chi-square test | Not mentioned | No Association |
|  | TRAV4 / rs1997533 | 14q11.2 | Briseño-Ruiz et al., 2013 [33] | Case Control / Turkey / 3 to 5 years | 172 - Caries free = 82; Caries experience (dmft > 4) = 90 | CC/CG/GG - 37/40/ 27 Vs 15/32/37 | C/G - 114/94 Vs 62/106 | Chi-square, Fisher’s exact and logistic regression. Allele (Model free) - OR (95% CI) = 0.48 (0.31 -0.73) Recessive Model - OR (95% CI) = 0.47 (0.26 - 0.95) | Genotype - p = 0.07; Allele (Model free) - p = 0.0005; Recessive Model - p =0.01; | G allele associated with low caries experience |
|  |  |  | Weber et al., 2018 [36] | Cohort / Norway / 5 years | 876; No Caries - 647; With Caries (low caries + high caries) - 228 | Not mentioned | C/G - recessive model - 4/93 Vs 61/528 | Plink Software - genotypic (2df) Test. Chi-Square test - χ2 = 3.772 | P = 0.05 | No Association |
| 32 | Human Leukocyte Antigen HLADRB1*04 - DR4  HLADQB1*02- DQ2  HLADQB1*03 -DQ3  HLADQB1*05- DQ5  HLADQB1*06 -DQ6 | 6p21.32 | Bagherian et al., 2008 [17] | Cross sectional / Iran / 12 - 71 months | 79; No caries - 35; ECC group - 44 | DR4 Allele - 0409/0403 - most common; Other genotypes not mentioned | Positive /Negative - Cases Vs Controls - DR4 -10/34 Vs 1/ 34; DQ2 - 24/20 Vs 14/21; DQ3 - 20/24 Vs 17/18; DQ5 - 15/29 Vs 10/25; DQ6 -22/22 Vs 14/21 | Student’s t test; ChiSquare and Fisher’s Exact test; OR 95% CI OR 95 % CI - DR4 -10 (1.16 - 87.1); Chi Square χ2 - DQ2 -1.65; DQ3 - 0.076; DQ5 - 0.275; DQ6 - 0.71 | DR4 - 0.036; DQ2 -0.199; DQ3 - 0.783; DQ - 0.6; DQ6 - 0.37; | frequency of the DRB1*o4 allele was significantly increased in the ECC group. |

IHS - Iowa Head Start Cohort; IFS - Iowa Fluoride Study Cohort

**Supplementary Table 3 - Table of Characteristics of Excluded Studies**

| S.No | Author / Title | Reason for exclusion |
| --- | --- | --- |
| 1 | Duverger. O, Carlson, JC, Karacz, CM, et al. Genetic variants in pachyonychia congenita-associated keratins increase susceptibility to tooth decay. PLoS Genet.2918;14(1): e1007168 | Age not matched (6 -12 years) |
| 2 | Silva MJ, Kilpatrick NM, Craig JM, et al. Genetic and Early-Life Environmental Influences on Dental Caries Risk: A Twin Study. Pediatrics.2019;143(5):e20183499. | No Genotyping |
| 3 | Morelli T, Agler CS, Divaris K. Genomics of periodontal disease and tooth morbidity. Periodontol. 2000.2020 82:143-156 | Periodontitis - Different Study design |
| 4 | Shungin D, Haworth S, Divaris K, et al. Genome-wide analysis of dental caries and periodontitis combining clinical and self-reported data. Nat Commun.2019;10(1):2773. | ECC not studied. Traits studied - DMSS, DMFS and Periodontitis - Different age group |
| 5 | Kelly AM, Kallistova A, Küchler EC, et al. Measuring the Microscopic Structures of Human Dental Enamel Can Predict Caries Experience. J Pers Med.2020;10(1):5. | Age not matched (Av age 10.46 yrs) |
| 6 | Wang Q, Jia P, Cuenco KT, et al. Association signals unveiled by a comprehensive gene set enrichment analysis of dental caries genome-wide association studies. PLoS One. 2013;8(8):e72653. | Different study design. Gene Ontology study using bio-informatics on 3 - 12 yr old patients |
| 7 | Wang Q, Jia P, Cuenco KT, et al. Multi-dimensional prioritization of dental caries candidate genes and its enriched dense network modules. PLoS One.2013; 8(10):e76666. | Different Study design |
| 8 | Strömberg N, Esberg A, Sheng N, et al. Genetic- and Lifestyle-dependent Dental Caries Defined by the Acidic Proline-rich Protein Genes PRH1 and PRH2. EBioMedicine 2017;.26:38-46. | Age not matched (12 years) |
| 9 | Pang L, Zhi Q, Zhuang P, et al. Variation in Enamel Formation Genes Influences Enamel Demineralization In Vitro in a Streptococcus mutans Biofilm Model. Front Physiol.2017 ; 8:851. | Age not matched (13 - 18 years) |
| 10 | Bayram M, Deeley K, Reis MF, et al. Genetic influences on dental enamel that impact caries differ between the primary and permanent dentitions. Eur J Oral Sci 2015;.123(5):327-334. | Age not matched (Average. Age 8.8 + 2.5 years) |
| 11 | Eriksson L, Esberg A, Simon Haworth S, Holgerson PL, Johansson I. Allelic Variation in Taste Genes Is Associated with Taste and Diet Preferences and Dental Caries. Nutrients.2019;11:1491 | Age not matched (18 - 23 years), not tested for caries |
| 12 | Ergöz N, Seymen F, Koray Gencay K, et al. Genetic Variation in Ameloblastin Is Associated with Caries in Asthmatic Children. Eur Arch Paediatr Dent.2014;15(3):211–216. | Age not matched (6 - 12 years) |
| 13 | Wang X, Shaffer JR, Weyant RJ,et al Genes and their effects on dental caries may differ between primary and permanent dentitions. Caries Res.2020;44(3):277-284. | No SNPs studied |
| 14 | Shaffer JR, Wang X, McNeil DW, et al. Genetic susceptibility to dental caries differs between the sexes: a family-based study. Caries Res.2015; 49(2):133-140. | No genotyping and No SNPs Studied |
| 15 | Dawson DV. New Genes are Identified That may be Associated With Childhood Caries. J Evid Based Dent Pract.2012; 12:225-227 | Commentary |
| 16 | Cavallari T, Moyses ST, Samuel Jorge Moyses SJ, Werneck RI. KLK4 Gene and Dental Decay: Replication in a South Brazilian Population. Caries Res.2017; 51:240–243 | Age not matched ( >12 years) |
| 17 | Holla LI, Linhartova PB, Kastovsky J, et al. Vitamin D Receptor Taq I Gene Polymorphism and Dental Caries in Czech Children. Caries Res. 2017;51:7-11 | Age not matched (13 to15 years) |
| 18 | Li ZQ, Hu XP, Zhou JY, X.-D. Xie XD, Zhang JM. Genetic polymorphisms in the carbonic anhydrase VI gene and dental caries susceptibility. Genet Mol Res.2015;14 (2):5986 - 5993 | Age not matched (51.16 + 9.48 years) |
| 19 | Halusic AM, Sepich VR, Shirley DC, et al. Calcium and Magnesium Levels in Primary Tooth Enamel and Genetic Variation in Enamel Formation Genes. Pediatr Dent.2014;36:384 - 388 | In-Vitro Study |
| 20 | Duverger O, Ohara T, Shaffer JR, et al Hair keratin mutations in tooth enamel increase dental decay risk. J Clin Invest.2014;124(12):5219-5224. | Age not matched (6 - 12 years) |
| 21 | Fine DH, Toruner GA Velliyagounder, K, Kumar VS, Dipti Godboley D, Furgang D. A Lactotransferrin Single Nucleotide Polymorphism Demonstrates Biological Activity That Can Reduce Susceptibility to Caries. Infect Immun.2013; 81(5):1596-1605 | Age not matched (Adults 28.8 to 38.6. years) |
| 22 | Shaffer JR, Wang X, Desensi RS, et al. Genetic susceptibility to dental caries on pit and fissure and smooth surfaces. Caries Res.2012;46(1):38-46. | No genotyping done. No SNPs tested |
| 23 | Werneck RI, Lázaro FP, Cobat A, et al. A major gene effect controls resistance to caries. J Dent Res.2011; 90(6):735-739. | Age not matched (Av Age - 30.72 yrs). No genotyping done |
| 24 | Ozturk A, Famili.P, Vieira. AR.The Antimicrobial Peptide DEFB1 Is Associated with Caries. J Dent Res.2010; 89(6):631-636 | Age not matched (17 to 84 years). |
| 25 | Dawson DV. Genetic factors appear to contribute substantially to dental caries susceptibility, and may also independently mediate sucrose sweetness preference. J Evid Based Dent Pract.2008; 8(1):37-39. | Commentary & Analysis article |
| 26 | Yu PL, Bixler D, Goodman PA, Azen EA, Karn RC. Human Parotid Proline-Rich Proteins: Correlation of Genetic Polymorphisms to Dental Caries. Genet Epidemiol.1986; 3(3):147-152 | Proteins studied |
| 27 | Fatturi AL, Menoncin BL, Reyes MT, et al. The relationship between molar incisor hypomineralization, dental caries, socioeconomic factors, and polymorphisms in the vitamin D receptor gene: a population-based study. Clin Oral Investig.2020; 24(11):3971-3980. | Age not matched (8 yrs) |
| 28 | Nicoline n, partakusuma fb, joenoes h, talbot c, auerkari ei. Association of enam c2452t polymorphism with high rates of caries occurrence in an indonesian population. Int J App Pharm.2020;12(1):1 | Age not matched (Adults) |
| 29 | Wang L, Li B, Tie X, Liu T, Zheng S, Liu Y. Association between HLA-DRB1* allele polymorphism and caries susceptibility in Han Chinese children and adolescents in the Xinjiang Uygur Autonomous Region. J Int Med Res.2019;48(4):300060519893852 | Age not matched (6 - 12 Yrs) |
| 30 | Vasconcelos KR, Arid J, Evangelista S, et al.. MMP13 Contributes to Dental Caries Associated with Developmental Defects of Enamel. Caries Res2019;.53(4):441-446. | Age not matched (10 to 12 yrs) |
| 31 | Olszowski T, Milona M, Janiszewska-Olszowska J, et al. The Lack of Association between FCN2 Gene Promoter Region Polymorphisms and Dental Caries in Polish Children. Caries Res.2017;51(1):79-84. | Age not matched (age > 15 yrs) |
| 32 | Dobrina K, Maria G, Ekaterina B, Vanyo M, Tanya K. Association study for the role of Matrix metalloproteinases 2 and 3 gene polymorphisms in dental caries susceptibility. J of Arch Oral Biol.2016; 68:9-12 | Age not matched (20 to 32 yrs) |
| 33 | Cogulu D, Onay H, Ozdemir Y, Aslan GI, Ozkinay, F, Eronat C. The Role of Vitamin D Receptor Polymorphisms on Dental Caries. J Clin Pediatr Dent.2016; 40(3):211-214 | Age not matched (6 - 12 Yrs) |
| 34 | Hu XP, Li ZQ, Zhou JY, Yu ZH, Zhang JM, Guo ML. Analysis of the association between polymorphisms in the vitamin D receptor (VDR) gene and dental caries in a Chinese population. Genet and Mol Res. 2015;14(3)11631-11638 | Age not matched. (Adults 30 - 67 yrs) |
| 35 | Wendell Sk, Wang X, Brown M, et al Genetic association of taste receptor pathways, caries and gender variations. 2012;The 81st Annual Meeting of the American Association of Physical Anthropologists | Conference Proceedings. Podium Abstract |
| 36 | Vieira AR, Bayram M, Seymen F, et al. In Vitro Acid-Mediated Initial Dental Enamel Loss Is Associated with Genetic Variants Previously Linked to Caries Experience. 2017. Front physiol. 8:104 | Subjects age not matched. Part of the study is InVitro |
| 37 | Sengul F, Kilic M, Gurbuz T, Tasdemir S.. Carbonic Anhydrase VI Gene Polymorphism rs2274327 Relationship Between Salivary Parameters and Dental-Oral Health Status in Children. Biochem Genet.2016; 54(4)467-475 | Age not matched (6 - 16 yrs) |
| 38 | Yarat A, Ozturk LK, Korkut Ulucan K, Aykuz S, Atala H, Isbir T. Carbonic anhydrase VI exon 2 genetic polymorphism in Turkish subjects with low caries experience (preliminary study). InVivo2011;:25(6):941-944 | Age not matched (18 to 26 yrs) |
| 39 | Letter to Editor. Might there be a link between mannose-binding lectin polymorphism and dental caries? Mol Immunol. 2005;42(9):1125-1127 | Age not matched (9.78 yrs) |
| 40 | Aribam VG, Aswath N, Ramanathan A. Single‑nucleotide in Vitamin D receptor gene and its association with dental caries in children. J Indian Soc of Pedod Prev Dent,2020; 38:8-13 | Age not matched (6 to 12 Yrs) |
| 41 | Akilpprienka B, Malarkodi M, Vivedharani R, et al.. Studies on the association of, DEFB1 gene polymorphism (rs11362 and rs1799946) and dental caries in South Indian population. J Datta Meghe Inst Med Sci Univ.2019; 14:237-40. | Age not mentioned - Samples from patients reporting to the dept. of Periodontics |
| 42 | Fine DH. Lactoferrin: A Roadmap to the Borderland between Caries and Periodontal Disease. 2015. J Dent Res.2015;94(6):768-776 | Review Article |
| 43 | Ohta M, Ohshima T, Nishimura H, Maeda N, Asada Y.. Mapping of a gene influencing initial dental caries susceptibility to chromosome 11. Pediatr Dent.2010; 20(1);84-90 | Study design different - Animal study |
| 44 | Saha R, Sood PB, Sandhu, Diwaker A, Upadhyaye S. Association of Amelogenin with High Caries Experience in Indian Children. J Clin Pediatr Dent. 2015;39(5):458-461. | SNPs not analysed |
| 45 | Orlova E, CarlsonJC, Lee MK, et al.Pilot GWAS of caries in African-Americans shows genetic heterogeneity. BMC Oral Health 2019; 19:215 | Data for children upto 6 years not available |
| 46 | Zeng Z, Feingold E, Wang X, et al. Genome-wide association study of primary dentition pit-and-fissure and smooth surface caries. Caries Res.2016; 48(4):330-338.. | Data for children upto 6 years not available |
| 47 | Shaffer JR, Wang X, Feingold E, et al. Genome-wide association scan for childhood caries implicates novel genes. J Dent Res.2011; 90(12):1457-1462. | Data for children upto 6 years not available |
| 48 | Wang X, Willing MC, Marazita ML, et al. Genetic and environmental factors associated with dental caries in children: the Iowa Fluoride Study. Caries Res.2012; 46(3):177-184. | Data for children upto 6 years not available |
| 49 | Divakar DD, Alanazi SAS, Assiri MYA, et al. Association between ENAM polymorphisms and dental caries in children. Saudi J Biol Sci.2019; 26:730-735 | Data for children upto 6 years not available |
| 50 | Kong YY, Zheng JM, Zhang WJ, The relationship between vitamin D receptor gene polymorphism and deciduous tooth decay in Chinese children. BMC Oral Health. 2017;17(1):111. | Data for children upto 6 years not available |
| 51 | Alyousef YM, Borgio JF, Azeez SA, et al. Association of MBL2 Gene Polymorphism with Dental Caries in Saudi Children. Caries Res.2017; 51:12-16 | Data for children upto 6 years not available |
| 52 | Antunes LS, Tannure PN, Antunes LA, et al. Genetic association for caries susceptibility among cleft lip and/or palate individuals. J Contemp Dent Pract.2014; 15(3):288-293. | Data for children upto 6 years not available |
| 53 | Chaussain C, Bouazza N, Gasse B, et al.. Dental caries and enamelin haplotype. J Dent Res. 2014;93(4):360-365. | Data for children upto 6 years not available |
| 54 | Tannure, P. N., Küchler, E. C., Falagan-Lotsch, P., Amorim, L. M., Raggio Luiz, R., Costa, M. C., et al. (2012a). MMP13 polymorphism decreases risk for dental caries. Caries Res.46(4), 401–407. | Polymorphisms of Immune response genes not analyzed |
| 55 | Gasse B, Grabar S, Lafont AG, et al.Common SNPs of AmelogeninX (AMELX) and dental caries susceptibility. J Dent Res.2013; 92(5):418-424. | Data for children upto 6 years not available |
| 56 | Vieira AR, Marazita ML, Goldstein-McHenry T. Genome-wide Scan Finds Suggestive Caries Loci. J Dent Res.2008; 87(5):435-439 | Data for children upto 6 years not available |
| 57 | Nazaryan R. Iskorostenskaya O., Gorenskaya O, Volkova N, Gargin V. Interrelation Of The Gene Cdkn1a (Rs 1801270) Polymorphic State And Level Of Development Of Caries In Children With Down Syndrome. Georgian Med News.2018; 2(275):112-1116 | Data for children upto 6 years not available |
| 58 | Shimomura-Kuroki J, Nashida T, Miyagawa Y, Sekimoto T. The Role of Genetic Factors in the Outbreak Mechanism of Dental Caries. J Clin Pediatr Dent.2018;42(1):32-36 | Data for children upto 6 years not available |
| 59 | Govil M, Mukhopadhyay N, Weeks DE, et al. Novel caries loci in children and adults implicated by genome-wide analysis of families. BMC Oral Health.2018;18(1):98. | Data for children upto 6 years not available |
| 60 | Meng, Wu T, Billings R, Kopycka-Kedzierawski DT, Xiao J. 2019. Human genes influence the interaction between Streptococcus mutans and host caries susceptibility: a genome-wide association study in children with primary dentition. Int J Oral Sci.11(2):19 | Data for children upto 6 years not available |
| 61 | Dodhia SA, West NX, Thomas SJ, et al. Examining the causal association between 25-hydroxy vitamin D and caries in children and adults: a two sample Mendelian randomization approach. Wellcome open Res. 2021;5:281 | Data for children upto 6 years not available |
| 62 | Alotaibi RN, Howe BJ, Chernus JM et al. Genome-Wide association study (GWAS) of dental caries in diverse populations. BMC Oral health 2021;21:377 | Data for children upto 6 years not available |
| 63 | Sanhueza J, Luis B, Rodriguez N, Borie-Echeverria E, Salinas P. Polymorphisms in DSSP (rs36094464) and RUNX2 (rs566712) Genes Contribute to the Susceptibility of Dental Caries in Childhood. Int J Morphol 2021;39(3):802 - 808 | Data for children upto 6 years not available |
| 64 | Wendell S, Wang X, Brown M, et al. Taste genes associated with dental caries. J Dent.Res. 2010 Nov;89(11):1198-1202. | Polymorphisms of Immune genes not analyzed |
| 65 | Aoki H, Imamura Y, Ouryouji K, Miyazawa H, Wang PL. Genetic polymorphism of the salivary mucin gene MUC7 in severe caries in Japanese pediatric patients. Pediatr Dent.J. 2010 ;20(2):152-157. | Polymorphisms of Immune response genes not analyzed |
| 66 | Anjomshoaa I, Briseño-Ruiz J, Deeley K, et al. Aquaporin 5 Interacts with Fluoride and Possibly Protects against Caries. PLoS One 2015;10(12):e0143068. | Polymorphisms of Immune response genes not analyzed |
| 67 | Ballantine JL, Carlson JC, Zandona AGF, et al. Exploring the Genomic Basis of Early Childhood Caries: A Pilot Study. Int J Paediatr Dent. 2018;28(2):217–225. | Polymorphisms of Immune response genes not analyzed |
| 68 | Katifelis H, Sioziou A, Gazouli M, Emmanouil D. ACTN2 (rs6656267) and MPPED2(rs11031093 and rs536007) polymorphisms in primary dentition caries: A case-control study. Int J Paediatr Dent. 2020;30(4):478–482. | Polymorphisms of Immune response not analyzed |
| 69 | Küchler EC, Pucinelli CM, Horta KC, et al. Dental Caries, Developmental Defects of Enamel and Enamel Microhardness Associated with Genetic Polymorphisms in the RANK/RANKL/OPG system. J Clin. Pediatr, Dent. 2020;44(1):35- 40. | Polymorphisms of Immune response genes not analyzed |
| 70 | Ohta M, Nishimura H, Asada. Association of DLX3 gene polymorphism and dental caries susceptibility in Japanese children. Arch Oral Biol. 2015;60(1):55-61 | Polymorphisms of Immune response genes not analyzed |
| 71 | Kastovsky J, Linhartova PB, Musilova K, et al. Lack of Association between BMP2 / DLX3 Gene Polymorphisms and Dental Caries in Primary and Permanent Dentitions. Caries Res. 2017;51(6):590–5. | Polymorphisms of Immune response genes not analyzed |
| 72 | Qin X, Shao L, Zhang L, Ma L, Xiong S. Investigation of Interaction between Vitamin D Receptor Gene Polymorphisms and Environmental Factors in Early Childhood Caries in Chinese Children. Biomed Res. Int. 2019:4315839. | Polymorphisms of Immune response genes not analyzed |
| 73 | Romanos HF, Antunes LS, Lopes LB, et al. BMP2 Is Associated with Caries Experience in Primary Teeth. Caries Res. 2015; 49(4):425–433. | Polymorphisms of Immune response genes not analyzed |
| 74 | Antunes LA, Machado CMC, Couto ACK, et al. A Polymorphism in the MTRR Gene Is Associated with Early Childhood Caries and Underweight. Caries Res. 2017;51(2):102–108 | Polymorphisms of Immune response genes not analyzed |
| 75 | Udina IG, Uchaeva VS, Volobuyev VV, Gracheva AS, Vasillev YY. Molecular Genetic Study of Association of the MTRR Gene A66G SNP with Dental Caries in Children with Congenital Cleft Lip and/or Palate and without Pathology. Russ J Genet. 2019;55(12):1577-1581. | Polymorphisms of Immune response genes not analyzed |
| 76 | Weber M, Hsin HY, Kalay E, et al. Role of estrogen-related receptor beta (ESRRB) in DFN35B hearing impairment and dental decay. BMC Med Genet.2014;15:81. | Polymorphisms of Immune response genes not analyzed |
| 77 | Eckert S, Feingold E, Cooper M, et al. Variants on chromosome 4q21 near PKD2 and SIBLINGs are associated with dental caries. J Hum Genet. 2017;62(4):491–496. | Polymorphisms of Immune response genes not analyzed |
| 78 | Olszowski T, Adler G, Janiszewska-Olszowska J, Safranov K, Chlubek D. DD Genotype of ACE I/D Polymorphism Might Confer Protection against Dental Caries in Polish Children. Caries Res. 2015;49(4):390–393. | Polymorphisms of Immune response genes not analyzed |
| 79 | Linhartova PB, Kastovsky J, Bartosova M, et al. ACE Insertion/Deletion Polymorphism associated with Caries in Permanent but not Primary Dentition in Czech Children. Caries Res. 2016;50(2):89–96. | Polymorphisms of Immune response genes not analyzed |
| 80 | Küchler EC, Deeley K, Ho B, et al. Genetic mapping of high caries experience on human chromosome 13. BMC Med Genet. 2013;14:116. | Polymorphisms of Immune response genes not analyzed |
| 81 | Kuchler EC, Feng P, Deeley K, et al. Fine mapping of locus Xq25.1-27-2 for a low caries experience phenotype. Arch Oral Biol. 2014;59(5):479–486. | Polymorphisms of Immune response genes not analyzed |
| 82 | Abbasoğlu Z,. Bussaneli DG, Tanboğa I, et al. Fine-mapping of Xq25.1–27.2 Shows Association of Early Childhood Caries with Genetic variants depending on Dietary Habits, Protecting Children who drink Milk before going to bed. Caries Res. 2019;53(3):333–338. | Polymorphisms of Immune response genes not analyzed |
| 83 | Shimuzu T, Deeley K, Ruiz JB, et al. Fine-Mapping of 5q12.1–13.3 Unveils New Genetic Contributors to Caries. Caries Res. 2013;47(4):273–283 | Polymorphisms of Immune response genes not analyzed |
| 84 | Olatosi OO, Li M, Alade AA, et al. Replication of GWAS significant loci in a sub-Saharan African Cohort with early childhood caries: a pilot study. BMC Oral Health. 2021;21(1):274. | Polymorphisms of Immune response genes not analyzed |
| 85 | Borgio JF, Alsuwat HS, Alamoudi W, et al. Exome array identifies functional exonic biomarkers for pediatric dental caries. Comput Biol Med. 2022;141:105019. | Polymorphisms of Immune response genes not analyzed |
| 86 | Patir A, Seymen F, Yildirim M, et al. Enamel formation genes are associated with high caries experience in Turkish children. *Caries Res.*2008;42:394–400. | Polymorphisms of Immune response genes not analyzed |
| 87 | Ouryouji K, Imamura Y, Fujigaki Y, et al. Analysis of mutations in the amelogenin and the enamelin genes in severe caries in Japanese pediatric patients. Pediatr Den J. 2008;18(2):79-85. | Polymorphisms of Immune response genes not analyzed |
| 88 | Gerreth K, Zaorsk K, Zabel M, Boryesewicz-Lewicka M, Nowicki M. Chosen single nucleotide polymorphisms (SNPs) of enamel formation genes and dental caries in a population of Polish children. Adv Clin Exp Med. 2017;26(6):899–905. | Polymorphisms of Immune response genes not analyzed |
| 89 | Shimuzu T, Ho B, Deeley K, et al. Enamel Formation Genes Influence Enamel Microhardness Before and After Cariogenic Challenge. PLoS One 2012;7(9):e45022 | Polymorphisms of Immune response genes not analyzed |
| 90 | Slayton RL, Cooper ME, and Marazita ML. Tuftelin, Mutans Streptococci, and Dental Caries Susceptibility. J Dent Res. 2005; 84(8):711-714. | Polymorphisms of Immune response genes not analyzed |
| 91 | Shaffer JR, Carlson JC, Stanley BOC, et al. Effects of enamel matrix genes on dental caries are moderated by fluoride exposures. Hum Genet. 2015;134(2):159–167. | Polymorphisms of Immune response genes not analyzed |
| 92 | Gerreth K, Zaorska K, Zabel M, Boryesewicz-Lewicka M, Nowicki M. Association of ENAM gene single nucleotide polymorphisms with dental caries in Polish children. Clin Oral Invest. 2016;20:631–636. | Polymorphisms of Immune response genes not analyzed |
| 93 | Linhartova PB, Deissova T, Musilova K, et al. Lack of association between ENAM gene polymorphism and dental caries in primary and permanent teeth in Czech children. Clin Oral Invest. 2018 May;22(4):1873-7. | Polymorphisms of Immune response genes not analyzed |
| 94 | Antunes LA, Antunes LS, Kuchler EC, et al. Analysis of the Association Between Polymorphisms In MMP2, MMP3, MMP9, MMP20, TIMP1, and TIMP2 Genes With White Spot Lesions And Early Childhood Caries. Int J Paediatr Dent. 2016;26(4):310-319. | Polymorphisms of Immune response genes not analyzed |
| 95 | Linhartova PB, Deissova T, Kukletova M, Holla LI. Matrix metalloproteinases gene variants and dental caries in Czech children. BMC Oral Health. 2020;20(1):138. | Polymorphisms of Immune response genes not analyzed |
| 96 | Lewis DD, Shaffer JR, Feingold E, et al. Genetic Association of MMP10, MMP14, and MMP16 with Dental Caries. Int J Dent. 2017:8465125. | Polymorphisms of Immune response genes not analyzed |
| 97 | Tannure PN, Küchler EC, Lips A et al. Genetic variation in MMP20 contributes to higher caries experience. J Dent. 2012(b);40(5):381-386. | Polymorphisms of Immune response genes not analyzed |
| 98 | Filho AVA, Calixto MS, Deeley K, Santos N. Rosenblatt A, Vieira AR. MMP20 rs1784418 Protects Certain Populations against Caries. Caries Res.2017;51(1):46–51. | Polymorphisms of Immune response genes not analyzed |
